# Design, verification and validation of a spatially confined magnetic nanoparticle thermal therapy device for conformal thermal dose contouring

**DOI:** 10.1080/02656736.2026.2717301

**Published:** 2026-08-19

**Authors:** Yash Sharad Lad, Shreeniket Pawar, Trent Townsend, Naveen Kondreddy, Anilchandra Attaluri

**Affiliations:** Department of Mechanical Engineering, School of Science, Engineering, and Technology, The Pennsylvania State University Harrisburg, Middletown, PA, USA

**Keywords:** Spatially confined-magnetic nanoparticle thermal therapy (SC-MNTT), field-free region, thermal dose (CEM43) painting, PID control, LabVIEW, phantom validation

## Abstract

**Background:**

Magnetic nanoparticle thermal therapy (MNTT) can localize heat within tumors, but non-uniform nanoparticle distribution can limit thermal dose coverage. Spatially confined MNTT (SC-MNTT) addresses this limitation by using a static magnetic field (SMF) to suppress heating outside a selected field-free region (FFR). However, SC-MNTT platforms require improved FFR steering and closed-loop temperature control.

**Purpose:**

This study developed and validated a low-cost, four-permanent-magnet SC-MNTT platform for spatially selective heating, dynamic FFR steering, and controlled thermal dose delivery in phantom models.

**Methods:**

The device combined four permanent magnets, an induction coil, motion and heating control, fiber-optic thermometry, and autotuning proportional-integral-derivative (PID) control. SMF maps and alternating magnetic field (AMF) defined operating conditions. Three-vial verification experiments evaluated spatially selective heating under pulsed AMF exposure. Liver-phantom validation experiments tested pulsed heating and autotuning PID control using prescribed temperature setpoint, a CEM43 target of 8 [min], and a healthy-tissue limit of 39.50 [°C].

**Results:**

The four-magnet configuration shifted the FFR between selected targets and concentrated heating at the intended nanoparticle location. In three-vial experiments, the selected vial reached normalized heating intensity near 1.00, while non-target vials remained lower between 0.09–0.26, corresponding to selective heating with reduced off-target activation. In liver-phantom experiments, autotuning PID control maintained a mean target temperatures 43.44 ± 0.01 [°C], achieved prescribed CEM43 at selected tumor probes, and kept the designated healthy-tissue probe below the safety threshold. Mean overshoot remained below 1.1 [%].

**Conclusion:**

The four-magnet SC-MNTT platform enabled repeatable in-plane FFR steering, spatially confined heating, and closed-loop thermal dose delivery in phantom models.

## Introduction

Magnetic nanoparticle thermal therapy (MNTT) is a minimally invasive therapy to treat deep and/or unresectable tumors. In MNTT, magnetic particles (MPs) are delivered to the tumor and exposed to a radiofrequency alternating magnetic field (AMF) [1,2]. The particles generate localized heat through magnetic hysteresis losses [3–8]. However, MPs may accumulate in off-target structures, where AMF exposure can cause unintended heating [9–11]. Spatially confined MNTT (SC-MNTT) can reduce unintended heating by limiting heat generation to a selected region [12–17]. Because MP distribution within tumors is often non-uniform, selective activation of MP deposits using SC-MNTT may improve thermal dose contouring while reducing the risk of damage to adjacent normal tissue [9–11,18,19].

Current MNTT systems and conventional AMF applicators are primarily designed to deliver an AMF over a broad treatment region [20]. Although this approach can activate MPs within the field, the AMF itself is not easily shaped to conform to irregular tumor boundaries or to selectively avoid nearby off-target nanoparticle deposits. As a result, any particles located within the exposed AMF region may generate heat, making conformal thermal dose delivery difficult when MP distribution is heterogeneous. SC-MNTT addresses this limitation by superimposing a static magnetic field (SMF) defined FFR onto the broader AMF exposure region.

In SC-MNTT, heating is spatially restricted by applying a SMF together with the AMF [12–17]. A sufficiently strong SMF saturates MPs outside the field-free region (FFR) and suppresses hysteresis losses, whereas MPs within the low-field FFR remain responsive to the AMF and generate heat. Thus, effective SC-MNTT requires knowledge of MP distribution so that the FFR can be aligned with regions requiring dose.

Three-dimensional MP concentration can be measured using computed tomography (CT) or magnetic particle imaging (MPI) [9,21–24]. However, CT-based MP quantification can be limited by radiation exposure, image sensitivity, and the high iron loading required for sufficient contrast and resolution. Therefore, MPI and ultrasound-based techniques are emerging alternatives for MP localization and treatment guidance. MPI uses the nonlinear magnetic response of MPs to reconstruct three-dimensional particle-distribution maps. Ultrasound-based methods, including magnetomotive ultrasound, magneto acoustic imaging, and thermoacoustic or thermal ultrasound imaging, can provide acoustic contrast related to MP location or heating response [25–27].

However, MPI quantification can be limited at high MP concentrations, where high-signal regions may produce saturation, haze, or signal spillover artifacts that obscure nearby low-signal regions [28,29]. Therefore, MPI may require scanner-specific reconstruction and artifact-correction algorithms, including high-dynamic-range or model-based reconstruction approaches. Ultrasound-based approaches may complement CT and MPI because of their portability and real-time imaging capability, but further validation is needed before they can be used for quantitative three-dimensional MP mapping and treatment planning.

Pre-clinical SC-MNTT systems have been developed with designs that may allow future integration with MPI [16,17]. Such integration could enable quantitative particle mapping and thermometry within the same therapeutic framework. Although MPI is not currently incorporated into these systems, compatible device geometry and magnetic configuration may help move SC-MNTT toward image-guided treatment.

Despite these advances, current SC-MNTT platforms mainly provide spatial control of heating and still have limited temperature control over time. As a result, treatment delivery can remain operator-dependent and less reproducible when MP distribution varies. To address limited temporal regulation, we previously incorporated autotuning PID control into SC-MNTT, building on prior PID-based MNTT studies [30–32]. Combining autotuning PID control with spatially confined heating can reduce operator dependence and may improve treatment consistency.

Prior SC-MNTT devices have demonstrated off-target heating suppression in preclinical settings. Computational study at the pre-clinical scale has shown that steering the FFR within the tumor may improve intra-tumoral thermal dose distribution [19]. In these studies, heating is often assumed to occur within the full-width at half-maximum (FWHM) region of the magnetic field profile in two-permanent-magnet pre-clinical systems. However, two-magnet systems may provide limited thermal coverage in irregular or complex tumor geometries. Therefore, translation of SC-MNTT to human-scale dose painting will require more flexible magnetic configurations, such as additional or stronger magnets.

In this study, we developed a low-cost, four-permanent-magnet proof-of-concept platform to improve in-plane FFR steering and evaluate conformal thermal dose delivery in phantom tumor models. Although the magnets were weaker than those used in prior preclinical systems, the multi-magnet configuration improved spatial targeting and supports further development toward thermal dose contouring.

This paper makes the following contributions:

1. Demonstration of spatially selective heating and controlled thermal dose accumulation in phantom models while maintaining predefined safety limits in non-target regions.
2. Introduction of a four-permanent-magnet SC-MNTT architecture that enables independent adjustment of the x- and y-direction in-plane gradients.
3. Integration of dynamic FFR steering with autotuning closed-loop thermal control to achieve both spatial and temporal regulation of heating.

## Materials and methods

### Device design

A three-dimensional computer-aided design of the device was created in Siemens NX (version 2412, Siemens, Plano, TX, USA) prior to fabrication to verify clearances, stage travel, and coil-magnet alignment (Figure 1). The system comprises four permanent magnets mounted on stepper-motor-driven linear stages with lead screws, a water-cooled induction heating unit with a copper solenoid coil, and a recirculating chiller. The following sections describe the hardware and software subsystems.

### Hardware

#### Permanent magnets.

Four high-strength neodymium disk magnets (McMaster-Carr, Part #5862K195; grade N52) were used to generate the SMF. Each magnet measured 76.2 [mm] in diameter and 25.4 [mm] in thickness (3 [in] x 1 [in]), with a maximum operating temperature of 79 [°C], a rated pull force of 225 [kg] (495 [lbs]), and a surface flux density of 582 [mT]. Magnets were arranged as two opposed pairs along the x- and y-axes with all north poles facing each other to maximize repulsive force and establish a stable FFR in the coil bore (Figure 1), similar to previously reported repelling-magnet geometries used for SC-MNTT [12–17,33–36]. Assembly followed established safety protocols for rare-earth magnets, including tool-assisted handling, mechanical restraints, and separation from sensitive electronic equipment.

#### Stepper motors and lead screw.

Four StepperOnline NEMA-23 closed-loop stepper motors (Model #23E1K-12, StepperOnline, China), each driven by a closed-loop driver with resolution of 0.09° (StepperOnline, Model #CL57T), controlled the spacing between the magnets. Each motor was coupled to a 4 [mm]-pitch lead screw, providing fine control of magnet position. Requested travel (reported in [mm]) was converted to required motor revolutions using the 4 [mm] pitch, enabling millimeter-scale FFR shifts. Linear guides and end-of-travel limit switches constrained motion and prevented mechanical overtravel.

#### AMF power supply and coil.

An Ambrell EASYHEAT 0112 water-cooled induction heating system (Ambrell Corporation, Rochester, NY, USA; 1.2 [kW]) generated the AMF. A custom copper solenoid coil with an inner diameter of approximately 70 [mm] and length of 48 [mm] was fabricated. Due to mounting and fixture constraints, the inner diameter and length can vary within ±2 [mm]. Field-amplitude calibration, H [kA/m] versus supply voltage [V], and AMF uniformity were characterized using a two-dimensional magnetic field probe (AMF Life Systems, Auburn Hills, MI, USA) with radial and axial sensitivity factors of 0.96 (Figure S1 in Supplementary Information). Within a cylindrical region centered in the bore, the AMF varied by <10 [%] for amplitudes up to 13.04 [kA/m] at 210 ± 10 [kHz]. These measurements informed operating limits for both verification and validation experiments.

#### Recirculating chiller.

A ThermoFlex™ TF1400 recirculating chiller (Thermo Fisher Scientific, Waltham, MA, USA) provided coolant to the induction unit. The chiller delivers up to 1.4 [kW] cooling capacity at 60 [Hz] and was operated to maintain coil coolant temperature at 20 ± 1 [°C]. Stabilizing the coolant temperature reduced drift in coil impedance and AMF amplitude during extended runs, thereby improving repeatability of heating conditions.

#### IR thermometry.

An FLIR E8 Pro infrared camera (Teledyne FLIR, Wilsonville, OR, USA) measured sample surface temperatures in the three-vial verification experiments. The detector resolution and stated accuracy are 320 × 240 and ±2 [°C], respectively. Emissivity was set to 0.92 based on the vial material (polypropylene), and the reflected apparent temperature was set to ambient (25 [°C]). The camera was positioned approximately 279 [mm] (11 [in]) from the coil at ~0° to the horizontal to minimize parallax and maintain a consistent field of view.

#### Optical fiber thermometry.

Temperature measurements for validation experiments were obtained with fiber-optic probes (FOT-L-SD-C1-F1-M2-R1-ST, FISO Technologies, Ltd., Quebec, QC, Canada), consistent with prior temperature-feedback MNTT devices [30–32]. Each probe contains a single sensing element within a 1.7 ± 0.2 [mm] diameter optical fiber and provides ±1.0 [°C] accuracy, 0.1 [°C] (rms) resolution, and 25–100 [ms] response time. Signals were acquired using a FISO TMI4 unit (FISO Technologies, Ltd., Quebec, QC, Canada). The fiber-optic probes, DAQ, and FISO TMI4 unit were calibrated in a water bath against a calibrated RTD probe. The optical probes and RTD probe were placed in a beaker with sponge supports to maintain fixed probe positions. The water bath was held at each temperature setpoint for more than 2 [h] until RTD temperature variations were below 0.1 [°C]. Five temperature setpoints were tested. At each setpoint, 1000 readings were recorded from each optical probe and the RTD probe. The readings were averaged, and a linear calibration curve was fitted across the five setpoints. The slope was obtained from the fitted line, and the offset was calculated from the temperature difference between each optical probe and the RTD probe.

#### DAQ and controller.

An NI myRIO-1900 (National Instruments, Austin, TX, USA) provided real-time position control and AMF amplitude modulation, while an NI USB-6001 (National Instruments, Austin, TX, USA) acquired temperature signals. Separating control and acquisition hardware reduced common-ground interference between the induction supply, motors, and thermometry. The myRIO generated 4 [kHz] pulse-width-modulated (PWM) signals with a 0.5 [%] duty cycle to drive each stepper motor driver, enabling slow, precise magnet motion. Digital inputs monitored limit switches to enforce travel limits. Analog input channels recorded temperature measurements, and analog output channels (0–10 [V]) modulated the induction power-supply voltage. A hard-wired emergency-stop switch interrupted power to the induction unit for immediate shutdown.

### Software

Two software modules were implemented in NI LabVIEW: (i) motor position control for automated, dynamic FFR adjustment and (ii) heating control with user-selectable modes (continuous, pulsed, and autotuning PID). In the positioning workflow, the operator selects numbered heating regions on the coil map, the GUI generates the corresponding FFR coordinates (FFR1-FFR4), and the myRIO converts requested travel [mm] into PWM pulse trains to execute motion profiles. Heating is disabled while the magnets are in motion to prevent off-target exposure during FFR transitions.

For heating control, continuous mode applies a constant AMF amplitude, pulsed mode uses user-defined ON/OFF durations and a maximum AMF amplitude, and the autotuning PID mode requires temperature and CEM43 setpoints. A probe near healthy tissue can be designated with a temperature cap (e.g., <39.5 [°C]) to limit off-target exposure [37–39]. Safety interlocks monitor all temperature channels and terminate induction power if user-defined thresholds are exceeded at any probe. Real-time plots display fiber-optic probe temperatures, accumulated CEM43, and AMF output versus time. After each programmed pulsed-heating period or autotuning PID phase, the system automatically advances to the next FFR coordinate. The autotuning PID implementation follows relay-based identification and gain selection strategies used previously for SC-MNTT control [32,40,41]. Additional details are provided in the Supplementary Information.

### DOE for field mapping

A 3^4^ full factorial design was created in Minitab^®^ (Minitab, LLC, State College, PA) to map the SMF. The four factors corresponded to the four permanent magnets, each evaluated at three positions: 0 [mm], 22.5 [mm], and 50 [mm] from the coil center, giving 81 magnet-position configurations. For each configuration, SMF measurements were acquired over the treatment-plane grid, with limited axial sampling over ±9.5 [mm] around the coil center to verify field behavior near the finite experimental region. The primary field characterization was presented as x-y maps because all verification and validation targets were positioned within an approximate axial plane (± 5 [mm]) and the present proof-of-concept study focused on in-plane FFR steering. These SMF maps, along with AMF uniformity measurements, were used to identify operating regions that produced a well-defined low-field region within the AMF plateau while avoiding excessive gradients at the coil edges. The complete SMF and AMF datasets are provided in (Supplementary Information Figures S2-S11).

#### Coordinate registration and FFR localization.

Magnet positions were referenced to a home position in which the opposing magnet pairs were symmetrically aligned about the coil center. The sample coordinate system was registered to the coil-bore center using the mechanical fixture and probe grid. For each selected magnet configuration, the FFR was identified as the local minimum in measured SMF magnitude within the mapped grid. Commanded FFR locations were then linked to heating regions by matching the measured low-field region with the vial or fiber-probe location. This registration step defined the magnet-position commands used for verification and validation experiments.

#### Magnetic nanoparticles.

All verification and validation heating experiments used Synomag^®^-Dextran plain 70 [nm] iron oxide nanoparticles (Micromod Partikeltechnologie, GmbH, Rostock, Germany; LN: 31324104–01) suspended in water, as received. Synomag^®^ particles were selected because they showed strong combined performance for MPI detection and MNTT heating in prior figure of merit analysis [42]. The nanoparticle concentration was 10 [mgFe/mL].

#### Relationship between SMF magnitude and nanoparticle saturation.

The measured SMF maps were interpreted relative to the magnetic response of the Synomag^®^-Dextran plain 70 [nm] particles. In SC-MNTT, nanoparticle saturation should not be interpreted as a sharp binary transition between fully active and fully inactive particles. The manufacturer reports saturation magnetization at an applied field of *H* = 800 [kA/m], corresponding to approximately 1.0 [T] in air, whereas the measured SMF magnitudes in the present device were up to approximately 100 [mT] [43]. However, suppression of AMF-driven heating can begin well below the field used to define saturation magnetization because any non-zero SMF bias reduces the dynamic magnetic susceptibility available to the AMF. Published magnetometry for Synomag-D 70 [nm] reported a low-field magnetic-response FWHM of approximately 4.5 [mT], indicating that the particle response is strongly affected in the low-mT range [44]. Therefore, regions near the measured SMF minimum were treated as the AMF-responsive FFR, whereas regions with higher SMF magnitudes were expected to show progressively reduced AMF response and suppressed heating. Since a lot-specific SLP-versus-SMF calibration was not performed for the Synomag^®^-Dextran plain 70 [nm] particles used in this study, the measured SMF maps were used to define relative FFR locations rather than a sharp saturation threshold.

#### Verification setup.

For verification, temperatures were measured only with the IR camera (Figure 2a). The three nanoparticle vials were arranged in a triangular configuration within the coil bore, approximately 120° apart. FFR positions were referenced to a home position at which all magnets were symmetrically aligned. In the first heating configuration, the FFR was centered symmetrically (x_Right_ = 100 [mm], x_Left_ = 100 [mm], y_Top_ = 0 [mm], y_Bottom_ = 0 [mm]) so the FFR overlapped all three vials, allowing simultaneous heating in each. In the second configuration, the FFR was shifted vertically toward the top vial (x_Right_ = 122.5 [mm], x_Left_ = 122.5 [mm], y_Top_ = 0 [mm], y_Bottom_ = 45 [mm]) to confine heating to the top vial while minimizing exposure in the other two. In the third configuration, the FFR was offset laterally toward the right vial (x_Right_ = 100 [mm], x_Left_ = 145 [mm], y_Top_ = 45 [mm], y_Bottom_ = 0 [mm]) to heat only the right vial. In the fourth configuration, the FFR was mirrored to the left (x_Right_ = 145 [mm], x_Left_ = 100 [mm], yT_Op_ = 45 [mm], y_Bottom_ = 0 [mm]) to heat only the left vial.

Pulsed heating used a 33 [%] duty cycle for 8 [min] per configuration, with AMF pulses alternating between 0 and 13.04 [kA/m] at 210 ± 10 [kHz] [45]. Heating was disabled during each FFR change to prevent unintended heating outside the target. Each configuration was repeated in triplicate for statistical evaluation.

#### Pulsed Box-Lucas analysis.

In the three-vial verification experiments, temperature-time data at each vial location (P1-P3) were acquired from the IR camera and analyzed using a non-adiabatic PBL model [46]. Per-pulse specific loss power (SLP) was computed from the PBL parameters using the standard nonadiabatic relation, which embeds thermal losses, and avoids subjective initial-slope window selection [46]. Additional details of the pulse-by-pulse PBL fitting procedure, parameter search ranges, sample properties, and SLP calculation are provided in the Supplementary Information. Equal nanoparticle volumes were injected into all three vials. To minimize bias from small variations in loading or placement and isolate the effect of FFR positioning, the experiment-level SLP for each condition was normalized per vial by the maximum SLP observed at that vial across all runs, yielding a dimensionless heating intensity in the range 0–1.

#### Reporting of absolute and normalized heating metrics.

Absolute PBL-derived SLP values were calculated for each pulse and retained as engineering performance metrics. Normalized heating intensity was then calculated to compare spatial selectivity after accounting for small vial-to-vial differences in nanoparticle volume, placement, and optical measurement conditions. The normalized metric therefore reports relative FFR targeting performance, while the absolute SLP values report heating efficiency under each operating condition.

#### Validation setup.

*(i) Liver phantom:* Liver phantom was fabricated using stereolithography (SLA). The STL file was prepared in PreForm and exported as a .form file for printing on a Form 2 printer (Formlabs, Somerville, MA, USA). Durable V2 resin was selected, and the layer height was set to 0.10 [mm]. The model was oriented 30° about the x-axis, supports were auto-generated with a raft, and post-processing included removal from the build platform, a 20 [min] rinse in 91 [%] isopropyl alcohol, support trimming, and post-curing in sunlight for ≥60 [min] with manual rotation. Porosity was evaluated by a water leak test.

Durable V2 resin was selected because it enabled reproducible SLA fabrication of a mechanically stable liver-shaped phantom with fixed probe locations and repeatable nanoparticle placement. The material was selected for device-level validation rather than tissue-mimicking thermal behavior. Reported thermal properties for SLA resins indicate thermal conductivity on the order of 0.25–0.35 [W/m-K], and specific heat capacity of approximately 2.1 [kJ/kg-K]. In comparison, liver/tumor-like soft tissue has higher water content, tissue-dependent thermal conductivity near approximately 0.5 [W/m-K], higher specific heat capacity, and perfusion-mediated heat removal. Therefore, the phantom was used to evaluate FFR steering, spatial selectivity, and closed-loop dose tracking under repeatable conditions rather than to reproduce absolute *in vivo* heat transfer.

For validation experiments, four fiber-optic probes (P1-P4) were inserted into the liver phantom positioned concentrically inside the coil (Figure 2b). Probes P1-P3 were placed in three distinct tumor-mimicking nodules, and P4 was positioned in surrounding parenchyma to represent healthy liver while allowing detection of potential nanoparticle diffusion into normal tissue. To evaluate dynamic FFR steering while avoiding heating in healthy tissue, an equivalent nanoparticle volume (~0.30 [mL]) was injected at all four locations. FFR positions were referenced to the home position for all measurements.

Two heating cycles were evaluated. (i) Pulsed heating: a 33% duty cycle at a constant field amplitude of 10.87 [kA/m] for 8 [min] per configuration. Four configurations were tested. First, the FFR was positioned (xRight = 110 [mm], xLeft = 100 [mm], yTop = 0 [mm], yBottom = 0 [mm]) to heat only the three left-side locations (P1-P3). The FFR was then sequentially centered on P1 (xRight = 145 [mm], xLeft = 100 [mm], yTop = 45 [mm], yBottom = 0 [mm]), P2 (xRight = 145 [mm], xLeft = 100 [mm], yTop = 0 [mm], yBottom = 45 [mm]), and P3 (xRight = 130 [mm], xLeft = 130 [mm], yTop = 0 [mm], yBottom = 45 [mm]) to confine heating to the targeted site while minimizing exposure at the remaining probes.

(ii) Closed-loop temperature and CEM43 dose-control validation: In the autotuning PID experiments, fiber-optic temperatures were recorded at four probe locations. The phantom experiments were performed under controlled laboratory conditions near room temperature. Because the purpose of these experiments was to evaluate FFR steering, autotuning PID behavior, and CEM43-based dose-tracking logic rather than to reproduce full *in vivo* heat transfer, a constant baseline offset was applied for the controller-equivalent CEM43 analysis. This offset shifted the initial temperature to a simulated physiological baseline. The resulting baseline-adjusted temperatures were used only to evaluate controller behavior relative to the 43 [°C] CEM43 framework. The direct measured phantom temperatures were not intended to represent *in vivo* tissue temperatures, and the baseline-adjusted CEM43 values should be interpreted as control-system dose-tracking performance rather than direct *in vivo* thermal dose. Thermal dose, expressed as cumulative equivalent minutes at 43 [°C] (CEM43), is given in Equation (1),

(1) $$\mathrm{CEM}43=\int_{t=0}^{t=\mathrm{final}}B^{43{}^{\circ}C‐T(x,y,z,t)}\mathrm{dt}$$

where B is an empirical constant derived from cell-culture measurements and is set to 0.5 for *T*≥ 43 [°C] and 0.25 for *T* < 43 [°C] [38,39,47,48]. The controller was autotuned using P1 and then operated with a temperature setpoint of 43.5 [°C], a dose setpoint of 8 [min], and a maximum field amplitude of 13.04 [kA/m]. The FFR was sequentially aligned with P1 (x_Right_ = 145 [mm], x_Left_ = 100 [mm], y_Top_ = 45 [mm], yBottom = 0 [mm]), P2 (xRight = 145 [mm], x_Left_ = 100 [mm], y_Top_ = 0 [mm], y_Bottom_ = 45 [mm]), and P3 (x_Right_ = 130 [mm], x_Left_ = 130 [mm], y_Top_ = 0 [mm], y_Bottom_ = 45 [mm]), advancing to the next location once CEM43 = 8 [min] was achieved at the active probe.

Scenario 1 was repeated in triplicate. Scenario 2 was performed as an additional feasibility test to evaluate whether the healthy-tissue constraint could be reassigned to a different probe location.

A second scenario was tested in which P3 was designated as the healthy-tissue probe and P1, P2, and P4 were treated as tumor probes to assess how precisely the FFR can be controlled with the current device specifications. In both cases, heating was turned OFF during each FFR retargeting step to prevent unintended off-target heating. Safety thresholds were enforced at 70 [°C] for any location and 39.5 [°C] at the designated healthy-tissue probe, consistent with thermal-dosimetry guidelines [15,49,50]. The pulsed experiment and Scenario 1 were repeated three times, while Scenario 2 was an additional single feasibility run.

## Results

The results are organized to evaluate the SC-MNTT platform from system operation to heating performance. First, the LabVIEW graphical user interface (GUI) is presented to show how FFR locations, heating modes, safety limits, and real-time feedback were implemented. Next, three-vial phantom experiments are used to verify spatially selective heating and dynamic FFR retargeting. Finally, liver-phantom experiments are used to validate pulsed heating and autotuning PID control under temperature and CEM43-based safety constraints. Together, these results demonstrate the ability of the device to coordinate magnet motion, AMF delivery, temperature feedback, and thermal-dose monitoring during spatially confined heating.

### In-plane SMF gradient tunability of the four-magnet architecture.

Gradient estimates for the tested magnet configurations are provided in the Supplementary Information. Across the measured configurations, *G*_*x*_ ranged from 0.65 to 3.00 [T/m], *G*_*y*_ ranged from 0.70 to 3.06 [T/m], and the *G*_*x*_ / *G*_*y*_ ratio varied from 0.40 to 1.86. These results show that the four-magnet architecture can generate both approximately balanced gradients and anisotropic in-plane gradient profiles. This tunability is important because conventional two-magnet configurations primarily define the selection-field gradient along one dominant axis, whereas independent motion of four magnets allows the in-plane field profile to be adjusted in both x and y directions.

### Graphical user interface (GUI).

Figure 3 shows the LabVIEW GUI developed for device operation, data acquisition, and real-time monitoring. The FFR tab allows the user to select numbered target regions on the coil map and register the corresponding FFR coordinates for sequential heating locations. The temperature-control tab allows the user to select continuous, pulsed, or autotuning PID heating modes and define key safety settings, including maximum AMF output, safety temperature, healthy-tissue probe, healthy-tissue temperature limit, and CEM43 setpoint. During each experiment, the GUI displays real-time temperature, accumulated CEM43, and AMF output traces, enabling continuous monitoring of both heating response and controller behavior. The acquired temperature, AMF output, probe status, CEM43, and time-stamped control data were stored as Microsoft Excel (.xls) files and postprocessed further in MATLAB to generate the temperature profiles, CEM43 curves, normalized heating intensity, and PBL-based SLP estimates reported in the following sections.

### Verification.

Verification results are shown in Figures 4, S13 and S14 (Supplementary Information). Figure 4a shows the applied pulsed AMF amplitude. Figure 4b confirms a uniform baseline with no heating. With the FFR centered, all three vials heated with similar SLPs (Figure 4c). Shifting the FFR to the top vial increased heating at the selected vial and reduced heating at the two non-target vials (Figure 4d). Moving the FFR to the right or left yielded selective heating of the right (Figure 4e) and left (Figure 4f) vials, respectively, with minimal off-target heating. Figure 4b-g verify spatial selectivity and dynamic retargeting of the FFR under pulsed AMF at fixed amplitude.

### Pulsed Box-Lucas analysis.

Normalized heating intensities averaged across runs are presented in Figure 4h, and detailed IR temperature, PBL fits, and per-pulse SLP values are provided in the (Supplementary Information Figures S15-S20). Table 1 reports the heating intensity (normalized SLP) for each selected FFR (All, P1, P2, P3), probe (P1-P3), and run. When all three vials were selected to heat, intensities at P1-P3 were moderate (~0.4–0.6) and similar across runs, consistent with distributed heating. When P1 was selected, intensity at P1 remained near 1 in all three runs, whereas P2 and P3 were suppressed to ≈0.17–0.21. Selecting P2 produced the opposite pattern, with an intensity of one at P2 and ~0.20–0.23 (P1) and ~0.10–0.11 (P3). Selecting P3 yielded an intensity of one at P3 and reduced values at P1 (~0.18–0.26) and P2 (~0.09–0.12). These per-run values show that the FFR positioning reproducibly concentrates heating at the chosen vial while consistently suppressing off-target vials.

Across the three verification runs, the selected vial consistently showed the highest normalized heating intensity. The highest non-target intensity ranged from approximately 0.17 to 0.26, depending on selected FFR position, indicating strong but incomplete suppression of off-target heating. This residual heating is expected because the static-field gradient creates a finite transition region rather than a sharp boundary between active and saturated nanoparticles.

### Validation.

Validation results are shown in Figures S21-S23 (Supplementary Information) for pulsed heating and Figures 5, S24 and S25 (Supplementary Information) for autotuning PID control. Both methods achieved the control objective of heating the desired tumor location while minimizing heating in healthy tissue. The corresponding autotuning PID parameters and closed-loop performance metrics for the liver-phantom experiments are summarized in Table 2. Across the three repeated Scenario 1 runs, the autotuning PID yielded similar proportional gains (KP≈10–12 [V/°C]) with short integral times (T, < 0.2 [min]) and zero derivative action. Mean overshoot remained below 1.1 [%], and the mean steady-state temperatures were tightly clustered around the 43.5 [°C] setpoint (43 [°C] for P3 in runs 1–3).

The autotuning PID controller maintained the active tumor-designated probe near the prescribed temperature plateau while limiting heating at the designated healthy-tissue probe. In addition to overshoot and steady-state temperature, future reporting should include time to setpoint, time to CEM43 = 8 [min], maximum healthy-probe temperature, maximum healthy-probe CEM43, and time spent at the maximum AMF limit. These metrics better describe both controller performance and safety-margin behavior.

For the closed-loop PID/CEM43 validation experiments, repeatability was evaluated using run-level performance metrics rather than error bars on the temperature and CEM43 time histories. Because the autotuning phase was experimentally driven, the duration of autotuning, time to setpoint, and FFR switching times were not identical across runs. Therefore, pointwise averaging of CEM43 curves would compare different control states and may be misleading. Instead, Table 2 summarizes repeatability using the autotuned gains, mean overshoot, and mean steady-state temperature. Across the repeated Scenario 1 runs, the controller maintained mean steady-state temperatures near the setpoint and mean overshoot below 1.1 [%], indicating repeatable closed-loop regulation under the tested conditions.

In the first PID scenario (Figure 5a-c), after an initial autotuning phase to identify controller gains (Figure 5a), the tuned phase maintains constant temperature ~43–43.5 [°C] at P1-P3 as the FFR is steered between them (Figure 5b), whereas P4 stays several degrees cooler. The corresponding CEM43 curves (Figure 5c) show prescribed doses at the tumor-designated probes and substantially lower dose at P4.

In a second PID scenario (Figure 5d-f), the same autotuning protocol was applied, after which the controller again produced near-target plateaus and ≥8 [min] CEM43 at P1, P2, and P4, while P3 remained near baseline temperature with negligible thermal dose (Figure 5e,f). These validation experiments demonstrate that the device can steer heating to selected intraparenchymal targets while limiting exposure at a designated healthy-tissue location.

## Discussion

This study demonstrates that a four-magnet SC-MNTT platform can dynamically steer the FFR to localize nanoparticle heating while closed-loop control regulates temperature and CEM43 delivery. Unlike prior SC-MNTT systems that primarily demonstrated spatial confinement, the present platform integrates in-plane FFR steering, temperature feedback, and dose monitoring in a single phantom-based experimental system [16,32]. These results extend prior computational work on FFR dose painting by experimentally showing that the FFR can be moved between target regions to redistribute heating in multi-target phantom models [19].

Field characterization confirmed that the observed spatial selectivity was primarily governed by the SMF configuration rather than AMF non-uniformity. The FFR remained within a central region where AMF variation was less than 10 [%] (Figure S11). Therefore, differences in heating were mainly attributed to FFR position, SMF bias, and nanoparticle location. This distinction is important because SC-MNTT should localize heating through the selection field rather than unintended AMF variation.

The three-vial experiments showed that FFR steering can redistribute heating between nanoparticle deposits. When the FFR was aligned with a selected vial, the targeted vial approached normalized heating near unity, while non-target vials remained lower at approximately 0.17–0.26 for the nearest vial and 0.09–0.12 for the farther vial (Figure 4h). These results support spatial selectivity, but also show that heating suppression was incomplete. This residual heating is expected because finite SMF gradients create a transition region rather than a sharp active-saturated boundary, and heat conduction can spread energy from the active region [13–15]. Therefore, future treatment planning should account for residual heating outside the intended target.

Nanoparticle heating suppression should be interpreted as a graded function of SMF magnitude rather than a sharp saturation boundary. Although the manufacturer reports saturation magnetization at a higher applied field than the SMF levels measured in this device, AMF-driven heating can decrease at lower bias fields because increasing SMF reduces the dynamic magnetic susceptibility available to the AMF. This is consistent with the reported low-field response width of Synomag-D 70 [nm] particles. Therefore, the measured SMF maps define relative regions of heating activation and suppression rather than an abrupt on/off boundary. A lot-specific SLP-versus-SMF calibration for Synomag^®^-Dextran plain 70 [nm] particles will be needed to quantitatively relate SMF magnitude, heating suppression, and delivered thermal dose.

Compared with the previous two-magnet SC-MNTT platform (HYPER) and its later autotuning PID validation study, the present system should be interpreted as a complementary low-cost proof of concept rather than a definitive demonstration of superiority [16,32]. HYPER demonstrated well-quantified 3D FFR positioning, adjustable FFR volume, and *in vivo* temperature regulation, whereas the present system focuses on direct in-plane FFR redistribution using four independently positioned permanent magnets without moving the sample. A key distinction is that two-magnet configurations primarily define the selection-field gradient along one dominant axis, while the present four-magnet system can independently vary the x- and y-direction gradients. In the measured configurations, *G*_*x*_ ranged from 0.65 to 3.00 [T/m], *G*_*y*_ ranged from 0.70 to 3.06 [T/m], and *G*_*x*_ / *G*_*y*_ varied from 0.40 to 1.86. This range indicates that the system can generate both balanced and anisotropic in-plane gradient profiles, which may support more flexible FFR shaping for irregular target geometries. However, the observed selectivity remained finite, with spatial selectivity on the order of ~7 [mm]. Achieving narrower margin control near ~2 [mm] would likely require higher gradients, improved calibration, and stronger field-generation hardware [12].

The current study used SMF mapping and heating response to define usable FFR positions, but did not fully quantify commanded-position error, FFR size, repeat-positioning uncertainty, or the relationship between planned FFR location and delivered heating centroid. Although all four permanent magnets were the same model and grade, apparent asymmetry between opposing magnets can still appear in measured SMF maps because of small differences in magnet-to-measurement distance, mounting alignment, mechanical tolerance, probe-grid registration, and coordinate-system definition.

As a conservative Type B sensitivity estimate, a 5 [%] pull-force tolerance was assumed for each nominal 500 [lbf] magnet. Since magnetic pull force scales approximately with *B*^2^, this gives an estimated field uncertainty of 2.5 [%] per magnet. For representative local fields of 30–70 [mT] and gradients of 1–3 [T/m], the propagated four-magnet FFR-location uncertainty is approximately 0.5–3.5 [mm]. With an additional 5 [mm] placement uncertainty per magnet, the combined in-plane FFR uncertainty is approximately 5.0–6.1 [mm]. This corresponds to an approximate 95 [%] Type B uncertainty interval of ±9.8–12.0 [mm], which is consistent with the observed 1.2 [cm] FFR offset. However, this is not a measured confidence interval because no Type A repeated-position uncertainty analysis was performed.

Closed-loop temperature control added temporal regulation to the spatial control produced by the FFR. For a setpoint of 43.5 [°C], the autotuning PID controller maintained clear temperature plateaus at the active probe, with mean overshoot below 1.1 [%] (Figure 5). Treated sites reached CEM43 values greater than 8 [min], while the designated healthy-tissue location received negligible dose in the tested cases (Figure 5c,f). The liver-phantom experiments further showed that this spatial selectivity could be maintained in a multi-target geometry with equal nanoparticle volumes at all probe locations. Safety was implemented by disabling heating during FFR motion and enforcing temperature limits at the designated healthy-tissue probe. However, the healthy-tissue threshold of 39.5 [°C] was reached during some runs, requiring manual setpoint reduction from 43.5 [°C] to 43 [°C]. Future systems should therefore include supervisory safety logic to automatically adjust setpoint, duty cycle, AMF amplitude, dwell time, or FFR trajectory as non-target regions approach safety limits.

The Durable V2 resin phantom isolated device performance from biological variability, allowing evaluation of FFR steering, heating selectivity, and PID regulation under controlled conditions. However, the phantom was not intended to reproduce vascularized tissue heat transfer. The resin differs from liver/tumor tissue in thermal conductivity, heat capacity, and, most importantly, absence of perfusion. In addition, the validation experiments were performed near room temperature and used baseline-adjusted equivalent temperatures for CEM43 analysis. Therefore, the reported CEM43 should be interpreted as controller-equivalent dose-tracking performance rather than direct *in vivo* thermal dose. Future studies should use physiologically temperature-controlled perfusion phantoms, *ex vivo* tissues, and/or *in vivo* models to evaluate absolute thermal dose delivery under realistic boundary conditions.

Nanoparticle distribution will be a major factor in future translation. The present phantoms used controlled nanoparticle volumes to isolate device performance, whereas *in vivo* tumors will likely have heterogeneous MNP uptake, washout, and possible off-target accumulation. Such heterogeneity can change the local heating rate, alter the AMF power required to maintain a temperature setpoint, and produce non-uniform CEM43 accumulation. A PID controller can regulate temperature at the measured feedback location, but it cannot guarantee uniform thermal dose in unmeasured tumor regions when MNP concentration is spatially heterogeneous. One motivation for dynamic FFR steering and thermal dose painting is to overcome this limitation by selectively activating different nanoparticle regions according to the measured or estimated particle distribution [9–10]. Therefore, future SC-MNTT workflows should combine image-guided MP mapping, multi-point thermometry, three-dimensional temperature reconstruction, and adaptive or supervisory control strategies, such as fuzzy logic or model predictive control, to adjust FFR dwell time, AMF amplitude, and target sequencing during treatment. Similar automated thermal-therapy control frameworks have used temperature or thermal-damage feedback with supervisory logic to adjust energy delivery and treatment position, supporting the use of adaptive target sequencing in future SC-MNTT systems [51]. Gel-based carriers or *in situ* gelling formulations may also help stabilize MP concentration, reduce washout, and improve agreement between planned and delivered dose [52,53].

Image-guided planning will be important because FFR steering controls where particles are activated, but effective dose delivery depends on knowing where particles are located. Quantitative three-dimensional MPI maps registered to the magnet and coil geometry could guide FFR locations, AMF amplitudes, and heating times, while ultrasound-based methods may provide complementary localization or treatment-monitoring feedback during MNTT [10,18,25–27,37]. In future SC-MNTT workflows, these imaging inputs could be combined with advanced controllers such as model predictive control to adjust FFR position, AMF level, and duty cycle under temperature and dose constraints [33]. However, quantitative calibration will be required before ultrasound-based measurements can be used for FFR path planning or thermal dose control.

The current device should be interpreted as a proof-of-concept platform for in-plane FFR steering rather than complete volumetric FFR control. Although spatial control of heating is three-dimensional in practical applications, the present experiments intentionally positioned the nanoparticle targets within a common axial plane to test whether FFR steering in the treatment plane could redistribute heating between target regions while limiting non-target heating. Limited axial sampling showed that the field variation near the central axial region was less pronounced than the x-y variation used for steering, so the sample could be positioned near the coil center along z while FFR steering was performed in-plane. Future systems may use z-axis sample/magnet positioning to align the treatment plane with the central low-field region, but active volumetric steering would require additional field-generation degrees of freedom, such as multi-magnet or multi-coil selection-field systems. Such platforms have been explored, including triple-axis gradient-field designs for more isotropic FFR control, but they introduce substantial engineering, calibration, and safety complexity [54]. Therefore, future translation will require full 3D field characterization, active z-direction steering, and volumetric heating validation.

The present results also suggest a possible path toward larger systems. Human-scale MPI systems already use gradient and drive-field hardware to generate FFRs inside larger bores [55]. Recent MPI-integrable magnetic particle hyperthermia hardware has also demonstrated an adult-head RF coil designed for clinical MPI integration, with phantom verification, canine-cadaver MPI/MNTT workflow validation, and human-head treatment simulation [56]. Some head-sized MPI designs use bore diameters on the order of 18–25 [cm], which suggests that FFR-based field systems can be scaled beyond small phantoms. For SC-MNTT, a similar concept could be used: gradient coils would define the FFR, and a separate drive-field coil would provide the AMF for heating. From this viewpoint, the permanent-magnet device developed in this study can be viewed as a small-scale testbed for selection-field-based heating and control.

Scaling SC-MNTT to deep-seated human tumors will require field-generation strategies beyond direct geometric scaling of the present permanent-magnet device. Low-frequency and static magnetic fields can penetrate tissue, but producing a well-defined FFR at depth requires sufficient SMF magnitude and gradient across a larger anatomical bore. As magnet-to-target distance increases, permanent-magnet field strength and gradient decrease, which can broaden the FFR, reduce off-target heating suppression, and limit conformal thermal dose delivery. Therefore, human-scale systems may require larger or stronger magnets, electromagnet-assisted selection fields, or multi-coil gradient-field designs to generate a controllable FFR at clinically relevant depths. Human-scale translation will also require a separate safety analysis. The product of magnetic field amplitude and frequency must be evaluated with respect to the Atkinson criterion [57,58], and larger systems will require careful evaluation of specific absorption rate, peripheral nerve stimulation, coil heating, and actuator limits. Therefore, the current device should not be viewed as a directly scalable clinical system. Instead, it provides an experimental testbed for studying the physics, control, calibration, and safety logic needed for future SC-MNTT systems.

Structural analysis of the magnet-support system was also not performed, and future work should evaluate mechanical stability, alignment accuracy, and deformation during repeated motion. In addition, the current device is not modular, which limits rapid changes in magnet spacing, coil position, and phantom configuration. Also, the safety logic required manual setpoint reduction when the healthy-tissue threshold was approached, and future versions should automate this step.

Another limitation of the present validation experiments is that the liver phantom was tested near room temperature and did not reproduce physiological perfusion, tissue heterogeneity, or body-temperature boundary conditions. Therefore, the baseline-adjusted CEM43 analysis should be interpreted only as a controller-equivalent dose-tracking assessment. It demonstrates that the device can coordinate FFR steering, feedback control, and safety constraints, but it does not represent direct *in vivo* thermal dose. Future studies will use physiologically temperature-controlled phantoms and/or *in vivo* models to evaluate absolute thermal dose delivery under more realistic thermal boundary conditions.

Future work should improve both device calibration and treatment planning. MPI-based nanoparticle maps should be incorporated into planning algorithms to select FFR paths, AMF levels, and dwell times. More advanced controllers, such as model predictive control or data-driven thermal models, should be tested to regulate both temperature and CEM43 while enforcing safety limits [59]. Device development should also focus on modular magnet positioning, improved calibration, z-direction steering, and automated safety control.

In summary, the four-magnet SC-MNTT platform provides a controlled experimental system for FFR-steered nanoparticle heating with feedback control. The system showed spatially selective heating, stable temperature regulation, and controlled CEM43 delivery in phantom models. The results support the feasibility of combining FFR steering with closed-loop control for thermal dose painting. Overall, this work provides an experimental step between computational FFR dose-painting studies and future image-guided SC-MNTT systems.

## Conclusion

This study demonstrates a low-cost four-magnet SC-MNTT platform capable of dynamically steering the field-free region to achieve spatially selective magnetic nanoparticle heating in phantom models. The system combined permanent-magnet FFR steering, calibrated AMF delivery, fiber-optic thermometry, and closed-loop PID control to regulate temperature and CEM43 accumulation at selected targets while limiting heating at designated non-target locations. These results provide experimental support for combining spatial control of heat generation with temporal feedback control for conformal thermal dose delivery.

The present platform should be interpreted as a proof-of-concept testbed rather than a directly scalable clinical device. Future work will focus on full device-specific calibration between commanded magnet position, measured FFR location, nanoparticle heating response, and delivered thermal dose. Additional studies are needed in perfused phantoms, *ex vivo* tissues, and *in vivo* animal models to evaluate performance under vascular heat loss, heterogeneous nanoparticle distribution, and biological variability. Integration with medical imaging technologies, such as MPI thermometry, or ultrasound-based nanoparticle/temperature monitoring, will also be important for image-guided treatment planning, three-dimensional temperature reconstruction, adaptive control, and eventual clinical translation of SC-MNTT.

## Supplementary Material

Supp 1

Supp 2

Supp 3

## Figures and Tables

**Figure 1.**
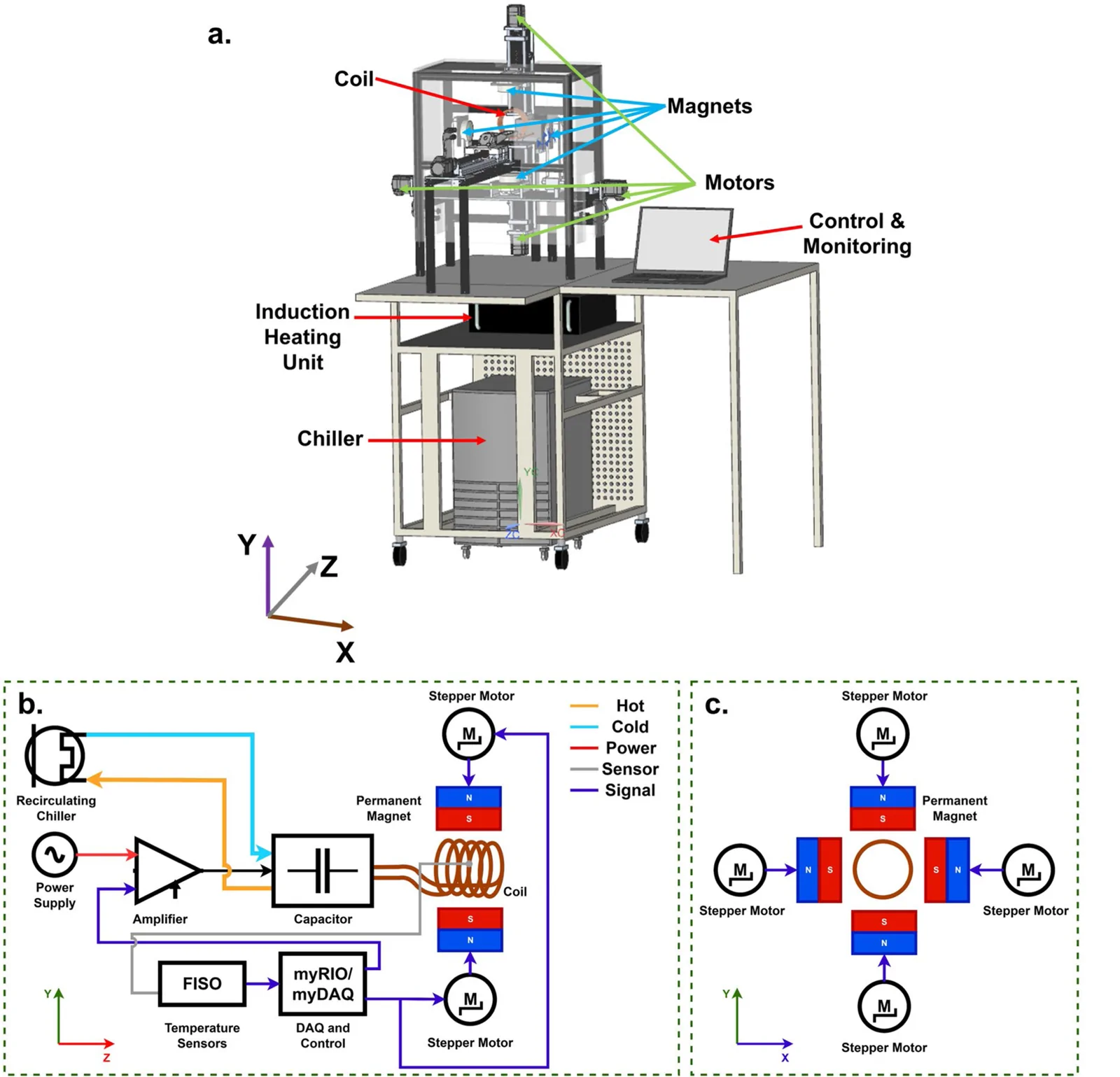
System design, architecture and magnet arrangement of the SC-MNTT device. (a) Three-dimensional computer aided design of the device was modeled prior to build using Siemens NX (version 2412). The system consisted of permanent magnets, stepper motors, a lead screw, an induction heating unit with copper coil, and a recirculating chiller. (b) Side view of the integrated system showing power supply, amplifier, capacitor, induction coil, permanent magnets, stepper motors, temperature sensors, and DAQ/control via NI myRIO and NI USB 6001. (c) Front view of the four-magnet configuration driven by stepper motors for in-plane FFR positioning.

**Figure 2.**
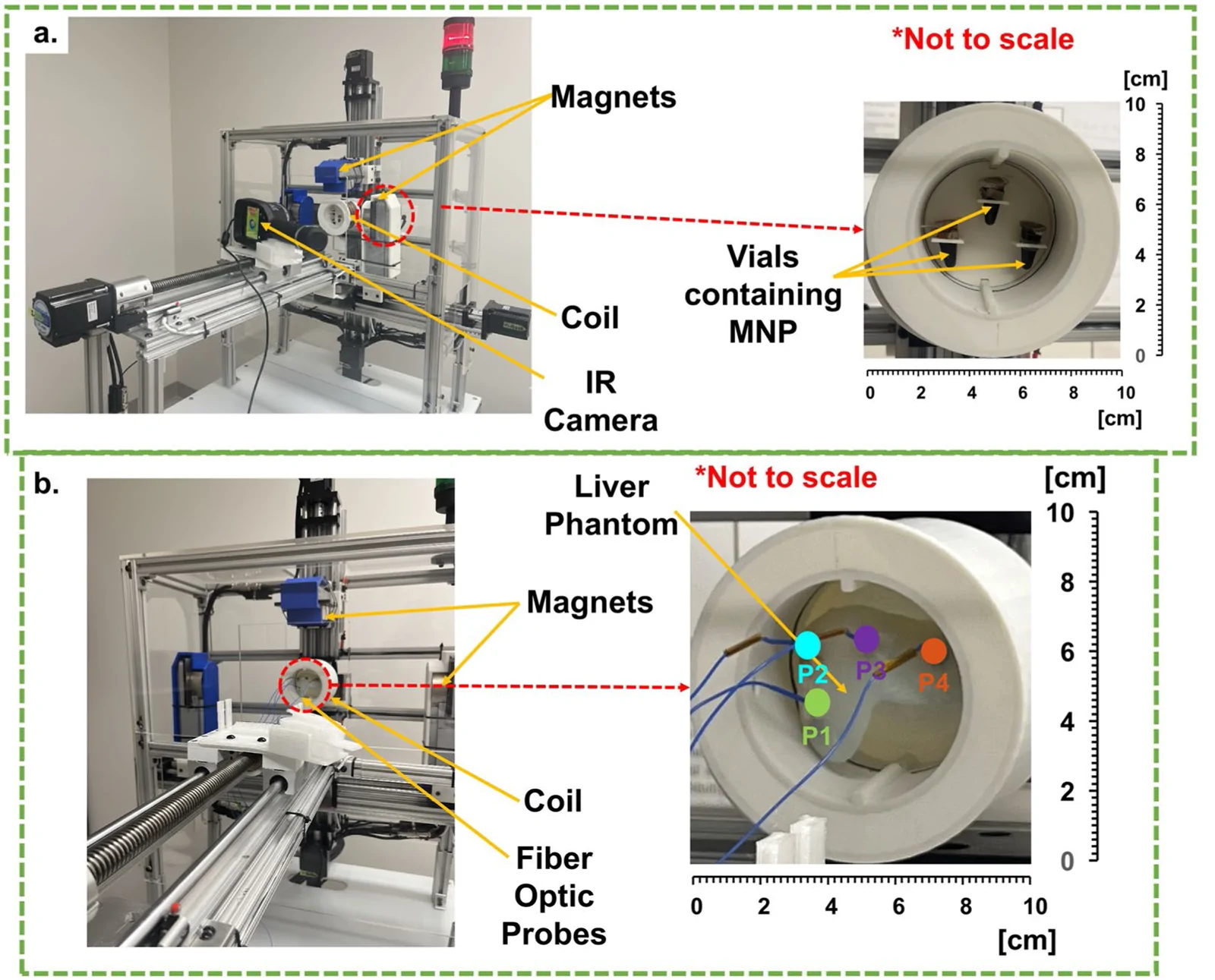
Experimental setup for verification and validation of spatially confined heating. (a) Verification: Magnet stage, Induction coil and an infrared camera for non-contact thermometry (on left). Coil bore showing three nanoparticle sample vials arranged ~120° apart (triangular configuration). Heating sequence: (1) all vials, (2) top vial, (3) right vial, (4) left vial (on right). (b) Validation: Magnet stage and induction coil with four fiber-optic temperature probes inserted into a 3D-printed liver phantom (on left). Right: Coil-bore view showing probe locations (P1-P4). P1-P3 were placed in tumor-mimicking nodules, P4 was placed in surrounding parenchyma to represent healthy tissue (on right). A schematic of the additively manufactured phantom is provided in the Supplementary Information as Figure S12, and a detailed engineering drawing is provided as a separate supplementary file.

**Figure 3.**
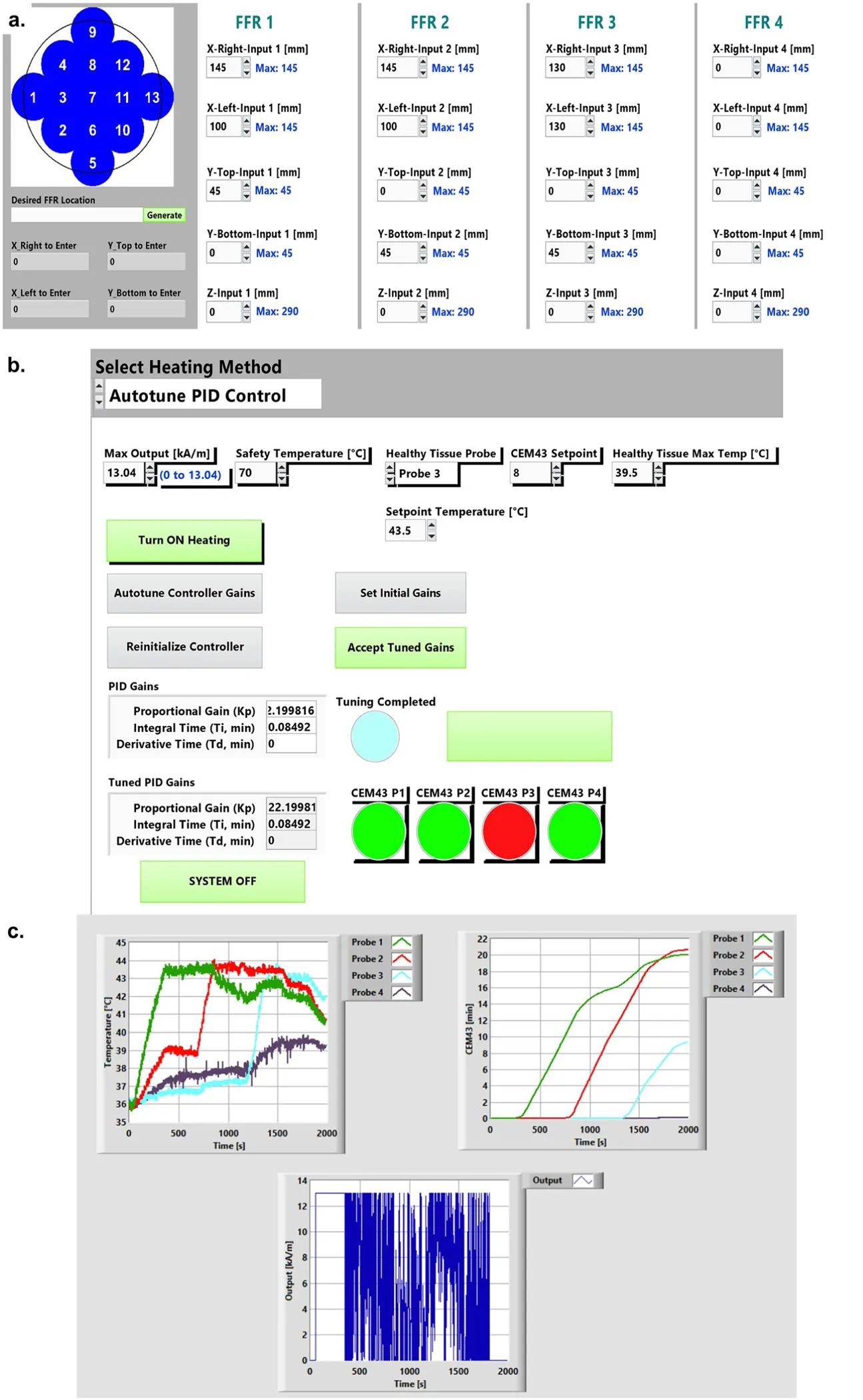
Graphical user interface for the device. (a) FFR tab on NI LabVIEW graphical user interface (GUI) for registering four target FFR locations prior to heating. User can select the numbered region to heat and obtain the FFR coordinates. These coordinates can be manually entered into the respective panels for FFR1, FFR2, FFR3, and FFR4 as X, Y, and Z inputs, with allowable travel limits. (b) and (c). Temperature tab on NI LabVIEW GUI for selecting heating mode. (b) User can select continuous, pulsed, and autotuning PID configurations, and specify safety constraints (maximum AMF output, safety temperature, healthy-tissue probe and maximum temperature, and CEM43 setpoint). (c) Real-time plots display fiber-optic probe temperature, accumulated CEM43, and AMF output versus time.

**Figure 4.**
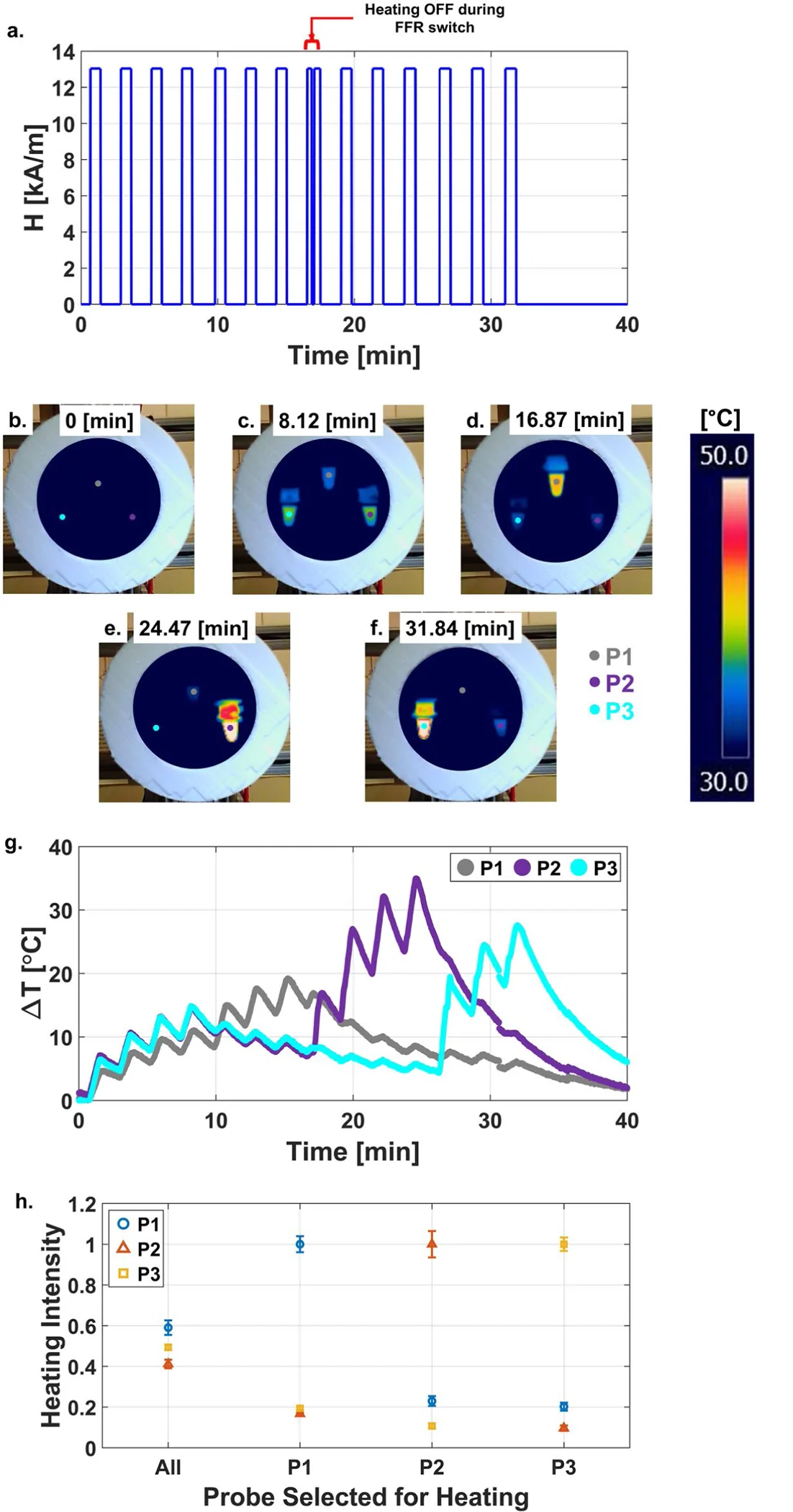
Verification of spatially selective heating with a steerable FFR. (a) Applied pulsed alternating magnetic field at 33 [%] duty cycle and fixed amplitude based on feedback from temperature and thermal dose. (b) Baseline IR image at *t*=0 [min] shows no temperature rise. (c) With the FFR centered to overlap all three vials, all three vials heat. (d-f) Steering the FFR to the top (d), right (e), and left (f) vial localizes heating to the targeted vial with only minimal off-target heating in the others. (g) Temperature versus time for regions of interest centered on each vial demonstrates pulsed heating and dynamic FFR steering. The temperature of the targeted vial rises during pulsed heating, while the non-targeted vials remain near baseline. This verifies that the device delivers controllable, spatially confined heating. (h) Normalized heating intensity derived from pulsed Box-Lucas analysis in the three-vial verification phantom at 33% duty cycle (13.04 [kA/m]). For each probe-selection condition on the x-axis (All, P1, P2, P3), the experiment-level SLP at each vial was normalized by the maximum SLP observed at that vial, yielding a dimensionless heating intensity between 0 and 1.

**Figure 5.**
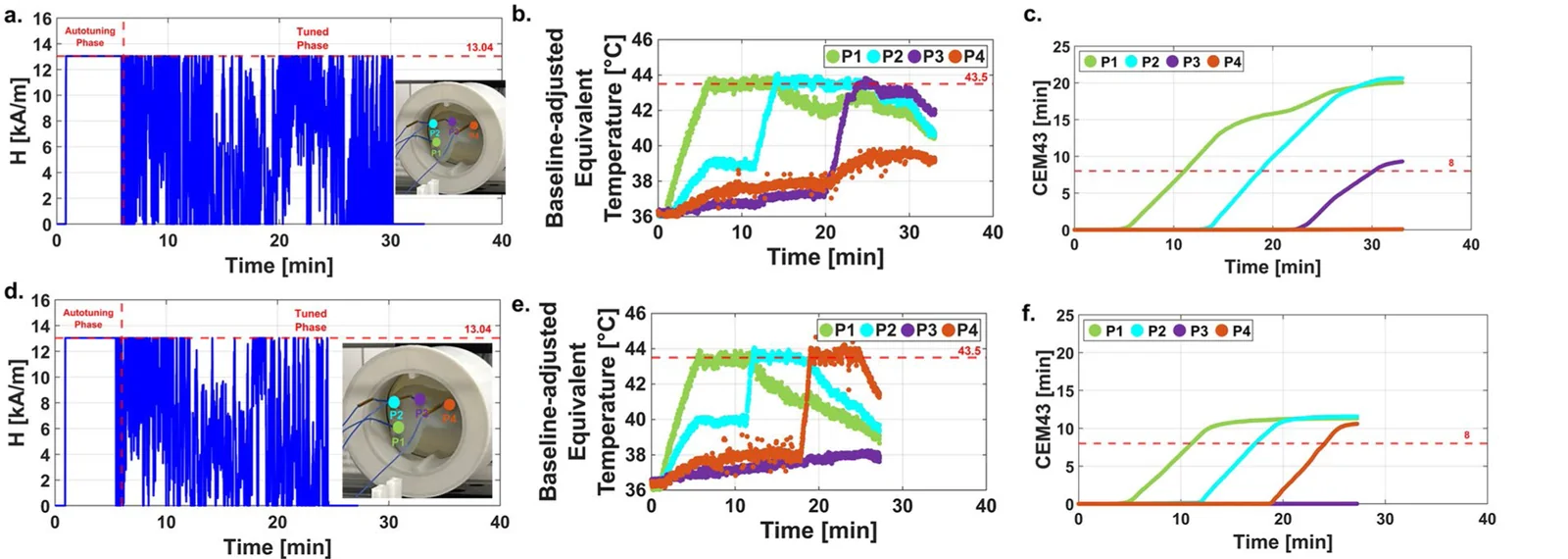
Autotuning PID validation in the liver phantom for two scenarios. (a-c) Scenario 1: P1-P3 tumor, P4 healthy tissue. (a) Applied AMF amplitude versus time, with the initial autotuning phase followed by the tuned phase; the dashed line indicates the maximum field amplitude of 13.04 [kA/m]. (b) Temperature trajectories at each probe: as the FFR is steered between targets, P1-P3 track plateaus near the 43.5 [°C] setpoint, whereas P4 remains several degrees cooler. (c) Corresponding CEM43 dose accumulation, demonstrating prescribed dose (>8 [min]) at P1-P3 and negligible dose at the healthy-tissue probe P4. (d-f) Scenario 2: P1, P2, and P4 tumor; P3 healthy. (d) Applied AMF amplitude versus time, with the initial autotuning phase followed by the tuned phase; the dashed line indicates the maximum field amplitude of 13.04 [kA/m]. (e) Temperature trajectories at each probe: during the tuned phase, P1, P2, and P4 are sequentially regulated near the 43.5 [°C] setpoint, whereas P3 remains below the 39.5 [°C] healthy-tissue limit. (f) Corresponding CEM43 dose accumulation, demonstrating prescribed dose (>8 [min]) at P1, P2, and P4 and negligible dose at the healthy-tissue probe P3. Temperature traces shown for the PID/CEM43 analysis represent baseline-adjusted equivalent temperatures used to evaluate controller dose-tracking from a simulated physiological baseline. These traces should not be interpreted as direct measured phantom temperatures. The phantom experiments were conducted near room temperature, and the baseline adjustment was used only for controller-performance evaluation.

**Table 1.** Normalized heating intensity for each selected FFR and vial (P1-P3) over three independent runs.

Selected	P1 heating intensity			P2 heating intensity			P3 heating intensity		
FFR	Run 1	Run 2	Run 3	Run 1	Run 2	Run 3	Run 1	Run 2	Run 3

All	0.59 ± 0.02	0.60 ± 0.04	0.55 ± 0.04	0.43 ± 0.01	0.41 ± 0.02	0.41 ± 0.03	0.49 ± 0.00	0.49 ± 0.01	0.51 ± 0.01
P1	1.00 ± 0.04	1.00 ± 0.01	1.00 ± 0.09	0.17 ± 0.00	0.17 ± 0.01	0.18 ± 0.01	0.19 ± 0.00	0.19 ± 0.01	0.21 ± 0.02
P2	0.23 ± 0.01	0.23 ± 0.02	0.20 ± 0.03	1.00 ± 0.06	1.00 ± 0.05	1.00 ± 0.06	0.10 ± 0.00	0.11 ± 0.01	0.11 ± 0.02
P3	0.26 ± 0.05	0.20 ± 0.02	0.18 ± 0.01	0.12 ± 0.03	0.09 ± 0.01	0.10 ± 0.00	1.00 ± 0.02	1.00 ± 0.08	1.00 ± 0.03

*Note*: to summarize spatial selectivity, the target-to-off-target suppression ratio was calculated as the selected-vial normalized heating intensity divided by the highest non-selected-vial intensity for each FFR position. Values are reported as mean ± standard deviation, where the standard deviation was calculated from the normalized PBL-derived SLP values obtained across the multiple heating pulses for each FFR condition, vial, and run.

**Table 2.** Autotuning PID gains and closed-loop performance for liver-phantom validation runs.

Runs	Tumor probes	Healthy tissue probe	KP [V/°C]	T_I_ [min]	T_D_ [min]	Setpoint [°C]	Mean overshoot [%]	Mean steady state temperature [°C]

1	P1, P2, P3	P4	12.01	0.06	0	43.5 (43 for P3)	1.00	43.46
2	P1, P2, P3	P4	12.03	0.05	0	43.5 (43 for P3)	0.59	43.42
3	P1, P2, P3	P4	10.01	0.19	0	43.5 (43 for P3)	0.96	43.44
4	P1, P2, P4	P3	22.20	0.08	0	43.5	0.73	43.45

## Data Availability

The data supporting the findings of this study are available within the article and its supplementary materials. Additional raw temperature, AMF, SMF mapping, and controller-output data are available from the corresponding author upon reasonable request.

## References

[1] Gilchrist RK, Medal R, Shorey WD, Selective inductive heating of lymph nodes. Ann Surg. 1957;146(4):596–606. doi: 10.1097/00000658-195710000-00007. PMC1450524 13470751

[2] Rosensweig RE. Heating magnetic fluid with alternating magnetic field. J Magn Magn Mater. 2002;252:370–374. doi: 10.1016/S0304-8853(02)00706-0.

[3] Krycka KL, Booth RA, Hogg CR, Core-shell magnetic morphology of structurally uniform magnetite nanoparticles. Phys Rev Lett. 2010;104(20):207203. doi: 10.1103/PhysRevLett.104.207203. 20867056

[4] Sousa EC, Rechenberg HR, Depeyrot J, In-field Mossbauer study of disordered surface spins in core/shell ferrite nanoparticles. J Appl Phys. 2009;106(9):093901. doi: 10.1063/1.3245326.

[5] Bakuzis AF, Morais PC, Pelegrini F. Surface and exchange anisotropy fields in MnFe 2 O 4 nanoparticles: size and temperature effects. J Appl Phys. 1999;85(10):7480–7482. doi: 10.1063/1.369383.

[6] Noh SH, Na W, Jang JT, Nanoscale magnetism control via surface and exchange anisotropy for optimized ferrimagnetic hysteresis. Nano Lett. 2012;12(7):3716–3721. doi: 10.1021/nl301499u. 22720795

[7] Margaris G, Trohidou K, Kachkachi H. Surface effects on the magnetic behavior of nanoparticle assemblies. Phys Rev B. 2012;85(2):024419. doi: 10.1103/PhysRevB.85.024419.

[8] Hu Y, Du A. The core-shell separation of ferromagnetic nanoparticles with strong surface anisotropy. J Nanosci Nanotechnol. 2009;9(10):5829–5833. doi: 10.1166/jnn.2009.1225. 19908460

[9] Attaluri A, Ma R, Qiu Y, Nanoparticle distribution and temperature elevations in prostatic tumours in mice during magnetic nanoparticle hyperthermia. Int J Hypertherm. 2011;27(5):491–502. doi: 10.3109/02656736.2011.584856. 21756046

[10] Carlton H, Arepally N, Healy S, Magnetic particle imaging-guided thermal simulations for magnetic particle hyperthermia. Nanomaterials. 2024;14(12):1059. doi: 10.3390/nano14121059. PMC11206764 38921935

[11] Healy S, Henderson ET, Ke S, Spatial analysis of nanoparticle distribution in human breast xenografts reveals nanoparticles targeted to cancer cells localized with tumor-associated stromal cells. Nanotheranostics. 2023;7(4):393–411. doi: 10.7150/ntno.84255. PMC10327423 37426881

[12] Tay ZW, Chandrasekharan P, Chiu-Lam A, Magnetic particle imaging-guided heating in vivo using gradient fields for arbitrary localization of magnetic hyperthermia therapy. ACS Nano. 2018;12(4):3699–3713. doi: 10.1021/acsnano.8b00893. PMC6007035 29570277

[13] Murase K A simulation study on the specific loss power in magnetic hyperthermia in the presence of a static magnetic field. OJAppS. 2016;06(12):839–851. doi: 10.4236/ojapps.2016.612073.

[14] Murase K, Takata H, Takeuchi Y, Control of the temperature rise in magnetic hyperthermia with use of an external static magnetic field. Phys Med. 2013;29(6):624–630. doi: 10.1016/j.ejmp.2012.08.005. 22985766

[15] Tasci TO, Vargel I, Arat A, Focused RF hyperthermia using magnetic fluids. Med Phys. 2009,36(5): 1906–1912. doi: 10.1118/1.3106343. PMC2736711 19544810

[16] Carlton H, Weber M, Peters M, HYPER: pre-clinical device for spatially-confined magnetic particle hyperthermia. Int J Hyperthermia. 2023;40(1):2272067. doi: 10.1080/02656736.2023.2272067. PMC10624165 37875265

[17] Buchholz O, Sajjamark K, Franke J, In situ theranostic platform combining highly localized magnetic fluid hyperthermia, magnetic particle imaging, and thermometry in 3D. Theranostics. 2024,−14(1 ):324–340. doi: 10.7150/thno.86759. PMC10750209 38164157

[18] Pawar S, Arepally N, Carlton H, Magnetic particle imaging resolution needed for magnetic hyperthermia treatment planning: a sensitivity analysis. Front Therm Eng. 2025,5:1520951. doi: 10.3389/fther.2025.1520951. PMC12037136 40303353

[19] Pawar S, Lad YS, Arepally N, Feasibility of thermal dose painting during magnetic nanoparticle thermal therapy: a computational analysis. Int J Hyperthermia. 2026,43(1):2635293. doi: 10.1080/02656736.2026.2635293. PMC13160377 41813594

[20] Rouni MA, Shalev B, Tsanidis G, A validated methodological approach to prove the safety of clinical electromagnetic induction systems in magnetic hyperthermia. Cancers (Basel). 2024,16(3):621. doi: 10.3390/cancers16030621. PMC10854696 38339373

[21] Johannsen M, Thiesen B, Gneveckow U, Thermotherapy using magnetic nanoparticles combined with external radiation in an orthotopic rat model of prostate cancer. Prostate. 2006,66(1 ):97–104. doi: 10.1002/pros.20324. 16114060

[22] Wust P, Gneveckow U, Wust P, Magnetic nanoparticles for interstitial thermotherapy-feasibility, tolerance and achieved temperatures. Int J Hyperthermia. 2006,22(8):673–685. doi: 10.1080/02656730601106037. 17390997

[23] Bruners P, Braunschweig T, Hodenius M, Thermoablation of malignant kidney tumors using magnetic nanoparticles: an in vivo feasibility study in a rabbit model. Cardiovasc Intervent Radiol. 2010,33(1):127–134. doi: 10.1007/s00270-009-9583-x. 19430744

[24] Goodwill PW, Conolly SM. Multidimensional x-space magnetic particle imaging. IEEE Trans Med Imaging. 2011,30(9):1581–1590. doi: 10.1109/TMI.2011.2125982. PMC3990467 21402508

[25] Hadadian Y, Uliana JH, Carneiro AA, A novel theranostic platform: integration of magnetomotive and thermal ultrasound imaging with magnetic hyperthermia. IEEE Trans Biomed Eng. 2021,68(1):68–77. doi: 10.1109/TBME.2020.2990873. 32356735

[26] Pi Z, Deng D, Chen X, Magneto-acoustic theranostic approach: integration of magnetomotive ultrasound shear wave elastography and magnetic hyperthermia. J Ultrasound Med. 2024,43(10):1819–1831. doi: 10.1002/jum.16512. 38872619

[27] Jansson T, Jansson L, Mousavi A, Detection of magnetomotive ultrasound signals from human tissue. Nanomedicine. 2023,47:102621. doi: 10.1016/j.nano.2022.102621. 36283571

[28] Sanders T, Carlton H, Korangath P, A field free line 3D reconstruction model for magnetic particle imaging for improved sensitivity, resolution, and high dynamic range imaging, 2025. arXiv Preprint. arXiv:2511.06627.

[29] Sanders T, Konkle J, Sehl OC, A physics-based computational forward model for efficient image reconstruction in magnetic particle imaging. IEEE Trans Med Imaging. 2025,44(5):2319–2329. doi: 10.1109/TMI.2025.3530316. PMC12203805 40031021

[30] Sharma A, Jangam AA, Yung Shen JL, Design of a temperature-feedback controlled automated magnetic hyperthermia therapy device. Front Therm Eng. 2023,3:1131262. doi: 10.3389/fther.2023.1131262. PMC10026551 36945684

[31] Sharma A, Jangam A, Shen JLY, Validation of a temperature-feedback controlled automated magnetic hyperthermia therapy device. Cancers (Basel). 2023,15(2):327. doi: 10.3390/cancers15020327. PMC9856953 36672278

[32] Pawar S, Carlton H, Lad YS, Verification and validation of an autotuning proportional integral derivative controller for spatially confined magnetic particle hyperthermia. J Med Device. 2026,20(2):021004. doi: 10.1115/1.4070364. PMC12755161 41479945

[33] Jian L, Shi Y, Liang J, A novel targeted magnetic fluid hyperthermia system using HTS coil array for tumor treatment. IEEE Trans Appl Supercond. 2012,23(3):4400104–4400104.

[34] Huang CF, Lin XZ, Lo WH. Design and construction of a hyperthermia system with improved interaction of magnetic induction-heating. 2010 Annual International Conference of the IEEE Engineering in Medicine and Biology. IEEE, 2010. p. 3229–3232. 10.1109/IEMBS.2010.5627199 21096603

[35] Makary MS, Khandpur U, Cloyd JM, Locoregional therapy approaches for hepatocellular carcinoma: recent advances and management strategies. Cancers (Basel). 2020,12(7):1914. doi: 10.3390/cancers12071914. PMC7409274 32679897

[36] Kotewall CN, Cheung TT. Microwave ablation for liver malignancy: a literature review. Clin Surg. 2016,1:1130.

[37] Lad YS, Pawar S, Arepally N, Thermal dose feedback control systems applied to magnetic nanoparticle hyperthermia. Int J Hyperthermia. 2025,42(1):2491519. doi: 10.1080/02656736.2025.2491519. PMC12129101 40289252

[38] Sapareto SA, Dewey WC. Thermal dose determination in cancer therapy. Int J Radiat Oncol Biol Phys. 1984,10(6):787–800. doi: 10.1016/0360-3016(84)90379-1. 6547421

[39] Dewhirst MW, Viglianti BL, Lora-Michiels M, Basic principles of thermal dosimetry and thermal thresholds for tissue damage from hyperthermia. Int J Hyperthermia. 2003,19(3):267–294. doi: 10.1080/0265673031000119006. 12745972

[40] Astrom KJ, Wittenmark B. Adaptive control, Dover publications. Mineola, New York: INC, 2008.

[41] Äström KJ, Hägglund T. Automatic tuning of simple regulators with specifications on phase and amplitude margins. Automatica. 1984,20(5):645–651. doi: 10.1016/0005-1098(84)90014-1.

[42] Carlton H, Salimi M, Arepally N, Ranking magnetic colloid performance for magnetic particle imaging and magnetic particle hyperthermia. Adv Funct Mater. 2025;35(2):2412321. doi: 10.1002/adfm.202412321. PMC11774450 39882193

[43] Synomag^®^ - micromod Partikeltechnologie GmbH. Micromod.De; 2017. https://micromod.de/produkt-kategorie/magnetische-partikel/synomag/.

[44] Vogel P, Kampf T, Rückert M, Synomag^®^: the new high-performance tracer for magnetic particle imaging. Int J Magn Part Imaging. 2021;7:1–6.

[45] Attaluri A, Kandala SK, Zhou H, Magnetic nanoparticle hyperthermia for treating locally advanced unresectable and borderline resectable pancreatic cancers: the role of tumor size and eddy-current heating. Int J Hyperthermia. 2020;37(3):108–119. doi: 10.1080/02656736.2020.1798514. PMC8363047 33426990

[46] Carlton H, Ivkov R. A new method to measure magnetic nanoparticle heating efficiency in non-adiabatic systems using transient pulse analysis. J Appl Phys. 2023;133(4):044302. doi: 10.1063/5.0131058. PMC9884152 36718210

[47] Connor WG, Gerner EW, Miller RC, Prospects for hyperthermia in human cancer therapy: part II: implications of biological and physical data for applications of hyperthermia to man. Radiology. 1977;123(2):497–503. doi: 10.1148/123.2.497. 322208

[48] Dewey WC, Hopwood LE, Sapareto SA, Cellular responses to combinations of hyperthermia and radiation. Radiology. 1977;123(2):463–474. doi: 10.1148/123.2.463. 322205

[49] Myrovali E, Maniotis N, Samaras T, Spatial focusing of magnetic particle hyperthermia. Nanoscale Adv. 2020;2(1):408–416. doi: 10.1039/c9na00667b. PMC9417684 36133972

[50] Dhavalikar R, Rinaldi C. Theoretical predictions for spatially-focused heating of magnetic nanoparticles guided by magnetic particle imaging field gradients. J Magn Magn Mater. 2016;419:267–273. doi: 10.1016/j.jmmm.2016.06.038. PMC5604258 28943706

[51] Pawar S, Lad YS, Arepally N, Automated laser retraction for targeted glioblastoma coverage during laser interstitial thermal therapy. Med Phys. 2026;53(2):e70267. doi: 10.1002/mp.70267. PMC12888946 41665525

[52] Abenojar EC, Wickramasinghe S, Ju M, Magnetic glycol chitin-based hydrogel nanocomposite for combined thermal and D-amino-acid-assisted biofilm disruption. ACS Infect Dis. 2018;4(8):1246–1256. doi: 10.1021/acsinfecdis.8b00076. 29775283

[53] Le Renard PE, Jordan O, Faes A, The in vivo performance of magnetic particle-loaded injectable, in situ gelling, carriers for the delivery of local hyperthermia. Biomaterials. 2010;31(4):691–705. doi: 10.1016/j.biomaterials.2009.09.091. 19878991

[54] Wang C, Li S, Zeng Z, A triple-axis hybrid-linearity selective gradient field system design in magnetic hyperthermia: providing a spheroidal shaped field-free region. J Appl Phys. 2026;139(8):085101. doi: 10.1063/5.0306597.

[55] Gräser M, Thieben F, Szwargulski P, Human-sized magnetic particle imaging for brain applications. Nat Commun. 2019;10(1):1936. 10.1038/s41467-019-09704-x PMC6486595 31028253

[56] Carlton H, Mattingly E, Mohtasebzadeh AR, Verification and validation in canine cadaver of an MPI-integrable RF head coil for magnetic particle hyperthermia. Int J Hyperthermia. 2026;43(1):2672017. doi: 10.1080/02656736.2026.2672017. PMC13541239 42270409

[57] Atkinson WJ, Brezovich IA, Chakraborty DP. Usable frequencies in hyperthermia with thermal seeds. IEEE Trans Biomed Eng. 1984;31(1):70–75. doi: 10.1109/TBME.1984.325372. 6724612

[58] Kumar A, Attaluri A, Mallipudi R, Method to reduce non-specific tissue heating of small animals in solenoid coils. Int J Hyperthermia. 2013;29(2):106–120. doi: 10.3109/02656736.2013.764023. PMC3991111 23402327

[59] Pawar S, Ghimirey B, Attaluri A, A compact microwave heating platform complemented by nonlinear model-based closed-loop temperature control. IEEE J Microw. 2025;6(2):540–549. doi: 10.1109/JMW.2025.3640415.

