# Supplementary material for: Design, verification and validation of a spatially confined magnetic nanoparticle thermal therapy device for conformal thermal dose contouring
Source: Int J Hyperthermia. Author manuscript; Available in PMC 2026 Oct 6. (PMC13639875; doi:10.1080/02656736.2026.2717301)
Supplement: Supplementary file 1 [file NIHMS2207194-supplement-Supp_1.pdf]

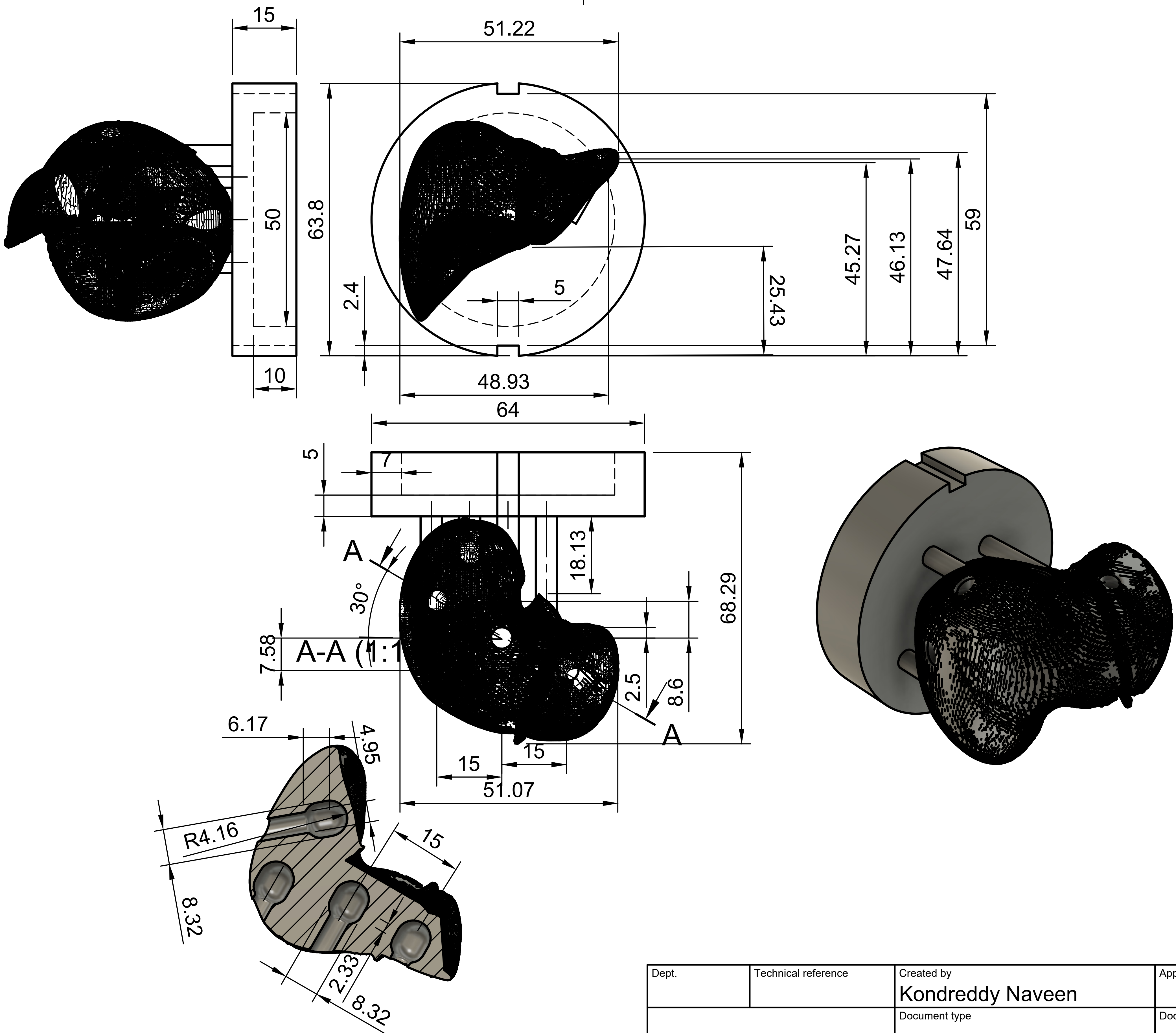

Dept.	Technical reference	Created by	Approved by
		Kondreddy Naveen	
		Document type	Document status
		Title: Validation Schematic	DWG No.
			Rev. / Date of issue / Sheet: 1/1
