# Supplementary material for: Design, verification and validation of a spatially confined magnetic nanoparticle thermal therapy device for conformal thermal dose contouring
Source: Int J Hyperthermia. Author manuscript; Available in PMC 2026 Oct 6. (PMC13639875; doi:10.1080/02656736.2026.2717301)
Supplement: Supplementary file 2 [file NIHMS2207194-supplement-Supp_2.pdf]

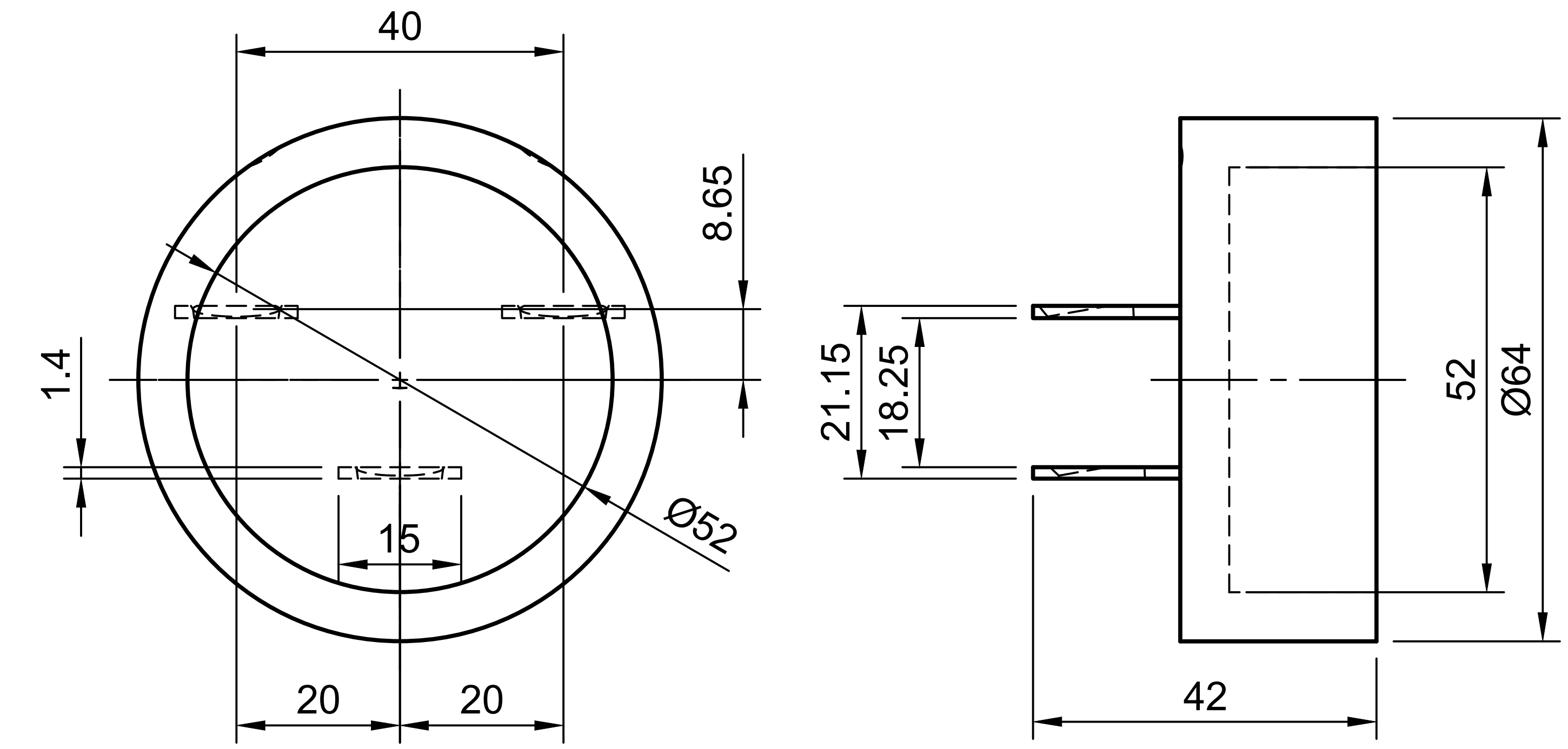


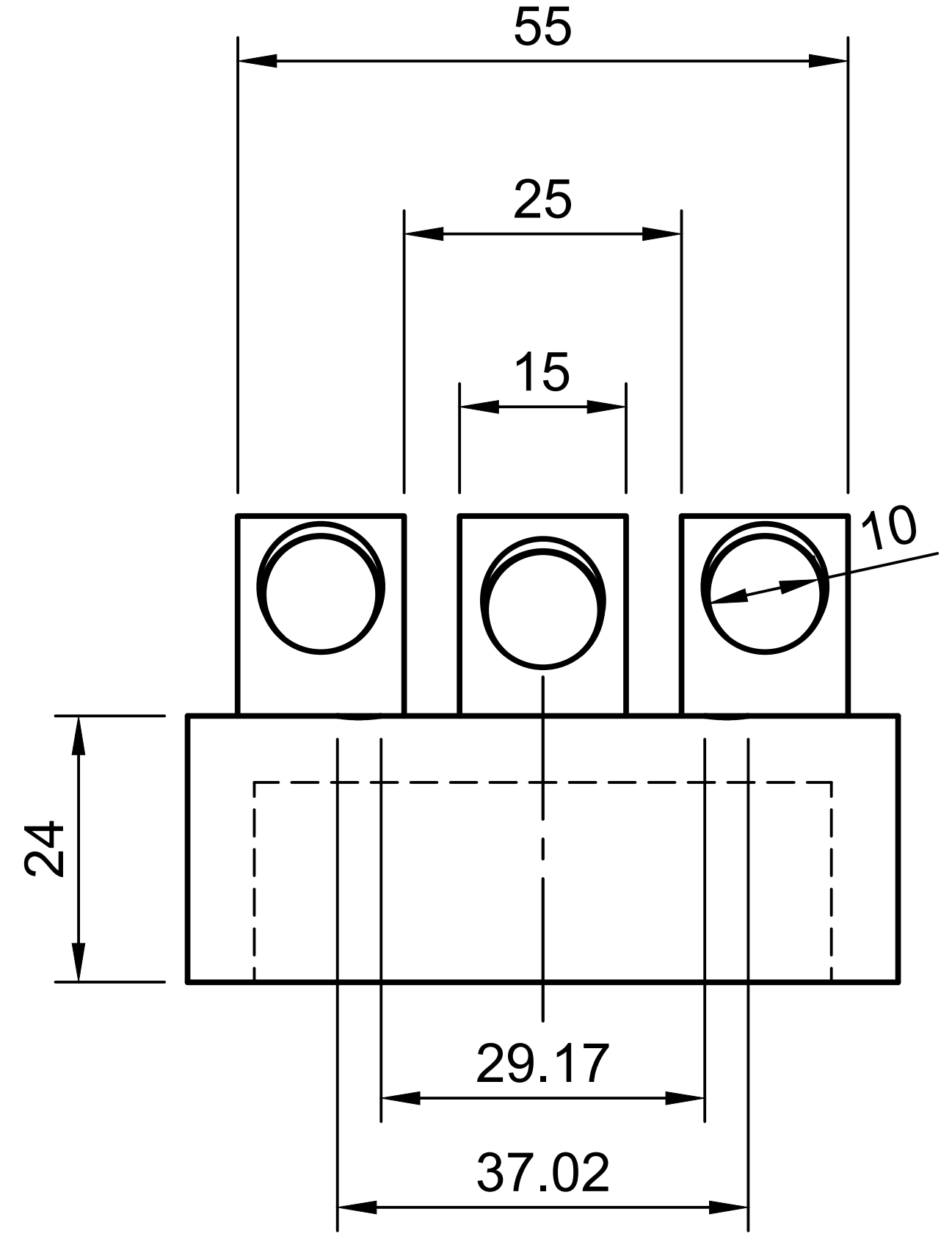


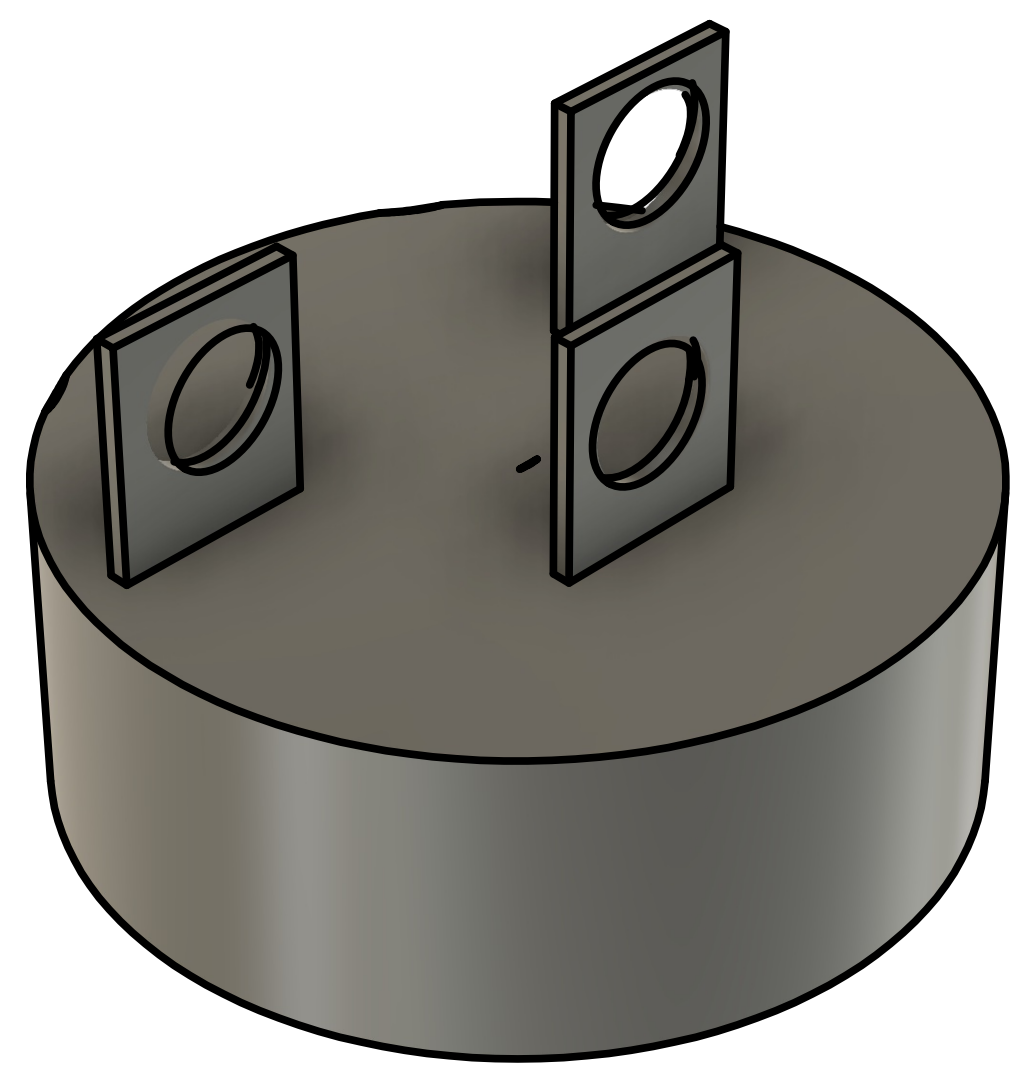

All dimensions are in millimeters (mm)

| Dept. | Technical reference | Created by | Approved by | | |
|---|---|---|---|---|---|
| | | Kondreddy Naveen | | | |
| | | Document type | Document status | | |
| | | Title<br>Verification Schematic | DWG No. | | |
| | | | Rev. | Date of issue | Sheet<br>1/1 |
