# Supplementary material for: Design, verification and validation of a spatially confined magnetic nanoparticle thermal therapy device for conformal thermal dose contouring
Source: Int J Hyperthermia. Author manuscript; Available in PMC 2026 Oct 6. (PMC13639875; doi:10.1080/02656736.2026.2717301)
Supplement: Supplementary file 3 [file NIHMS2207194-supplement-Supp_3.docx]

**Design, verification and validation of a spatially confined magnetic nanoparticle thermal therapy device for conformal thermal dose contouring**

*Authors: Yash Sharad Lad; Shreeniket Pawar^1^; Trent Townsend^1^; Naveen Konreddy^1^; Anilchandra Attaluri^1, *^*

**Affiliations:**

*^1^ Department of Mechanical Engineering, School of Science, Engineering, and Technology, The Pennsylvania State University Harrisburg, Middletown, PA, USA*

*^*^Correspondence:* [*aua473@psu.edu*](mailto:aua473@psu.edu) *(A.A.)*

**Autotuning PID controller**

During the initial autotuning phase, the controller output was switched in a relay/bang-bang manner to induce a controlled oscillatory temperature response. The resulting temperature response was used to estimate the ultimate period, $T_{u}$, and ultimate gain, $K_{u}$,. The PID controller was implemented as shown in Equation (S1):

| $U_{\mathrm{ctrl}}(t)=K_{c}\left( e(t)+\frac{1}{T_{i}}\int_{0}^{t} e(\tau)d\tau+T_{d}\frac{de(t)}{dt} \right),$ | (S1) |
| --- | --- |

with the autotuned parameters selected as shown in Equation (S2):

| $K_{c}=0.25K_{u}, T_{i}=0.5T_{u}, T_{d}=0.12T_{u}.$ | (S2) |
| --- | --- |

The ultimate gain was calculated from the relay response as shown in Equation (S3):

| $K_{u}=\frac{4U_{\max}}{\pi T_{a}},$ | (S3) |
| --- | --- |

where $U_{\max}$ is the maximum controller output and $T_{a}$ is the amplitude of temperature deviation from the setpoint during autotuning.

**Pulsed Box-Lucas analysis and SLP estimation**

Temperature-time data from the selected vial/probe during each pulsed-heating condition were analyzed using a non-adiabatic pulsed Box-Lucas (PBL) model [S1]. The lumped temperature rise was defined as shown in Equation (S4):

| $\theta(t)=T(t)-T_{\infty}$ | (S4) |
| --- | --- |

where $\theta(t)$ is the lumped temperature rise [°C], $T(t)$ is the measured sample temperature [°C], and $T_{\infty}$ is the ambient reference temperature [°C].

During each duty-cycle segment, heating and cooling were fit separately. The pulsed AMF condition used a 33 [%] duty cycle with 45 [s] heating and 90 [s] cooling. During heating, the temperature rise was fit as shown in Equation (S5):

| $\theta(t)=a+\left( \theta_{i} - a \right)e^{-b(t-t_{0})}$ | (S5) |
| --- | --- |

and during cooling as shown in Equation (S6):

| $\theta(t)=\theta_{i}e^{-b(t-t_{0})}$ | (S6) |
| --- | --- |

where $a$ is the asymptotic temperature rise [°C], $b$ is the lumped loss constant [1/s], $\theta_{i}$ is the segment initial temperature rise [°C], and $t_{0}$ is the segment start time [s]. Fits were performed pulse-by-pulse for the selected heating location. Non-selected probes were recorded for spatial selectivity analysis but were not used for SLP estimation.

For each heating segment, the non-adiabatic PBL model gives Equation (S7):

| $\frac{d\theta}{dt}=ab-b\theta$ | (S7) |
| --- | --- |

So, the product $ab$ represents the loss-corrected initial heating rate [°C/s]. The per-pulse SLP was calculated as shown in Equation (S8):

| $SLP=\frac{mc_{p}}{m_{\mathrm{Fe}}}ab$ | (S8) |
| --- | --- |

where $m$ is the total sample mass [g], $c_{p}$ is the specific heat capacity [J/(g·K)], and $m_{\mathrm{Fe}}$ is the iron mass [g_Fe_]. The following values were used: $\rho=1.0$ [g/mL], $c_{p}=4.18$ [J/(g·K)], nanoparticle concentration = 10 [mg_Fe_/mL], sample volume $V=0.35$ [mL], and $m_{\mathrm{Fe}}=V\times10/1000=0.0035$ [g_Fe_]. The total sample mass was calculated as shown in Equation (S9):

| $m=V\rho+\frac{V\times10}{1000}=0.3535\text{ [g]}$ | (S9) |
| --- | --- |

The Box-Lucas parameter search ranges were $a=0.1$ to 120 [°C] with 0.1 [°C] spacing and $b=0.0001$ to 0.02 [1/s] with 0.0001 [1/s] spacing. For each heating condition, the experiment-level SLP was reported as the arithmetic mean of the per-pulse SLP values, with the standard deviation used as uncertainty.

**Linear calibration of AMF amplitude to controller voltage:**

Figure S1: Calibration of AMF amplitude versus controller output voltage. Measured magnetic field amplitude H at the solenoid center is shown for stepped controller voltages. The solid red line depicts the linear least-squares fit. The dashed line shows the enforced zero-intercept model.

**Static magnetic field mapping:**

Figure S2: Measured SMF magnitude across the experimental grid, showing formation of the field gradient used to generate the FFR.

Figure S3: Measured SMF magnitude across the experimental grid, showing formation of the field gradient used to generate the FFR.

Figure S4: Measured SMF magnitude across the experimental grid, showing formation of the field gradient used to generate the FFR.

Figure S5: Measured SMF magnitude across the experimental grid, showing formation of the field gradient used to generate the FFR.

Figure S6: Measured SMF magnitude across the experimental grid, showing formation of the field gradient used to generate the FFR.

Figure S7: Measured SMF magnitude across the experimental grid, showing formation of the field gradient used to generate the FFR.

Figure S8: Measured SMF magnitude across the experimental grid, showing formation of the field gradient used to generate the FFR.

Figure S9: Measured SMF magnitude across the experimental grid, showing formation of the field gradient used to generate the FFR.

Figure S10: Measured SMF magnitude across the experimental grid, showing formation of the field gradient used to generate the FFR.

Table S1: SMF gradient and eccentricity for all the configurations.

| **X Left [mm]** | **X Right [mm]** | **Y Top [mm]** | **Y Bottom [mm]** | **G_x_ [T/m]** | **G_y_ [T/m]** | **G_x_/G_y_** | **R^2^** |
| --- | --- | --- | --- | --- | --- | --- | --- |
| 108.5 | -63.5 | 63.5 | -108.5 | 1.64 | 1.21 | 1.36 | 0.91 |
| 108.5 | -108.5 | 86 | -108.5 | 2.80 | 2.25 | 1.24 | 0.96 |
| 63.5 | -63.5 | 86 | -108.5 | 0.96 | 1.51 | 0.64 | 0.96 |
| 63.5 | -108.5 | 63.5 | -63.5 | 1.19 | 0.83 | 1.44 | 0.91 |
| 63.5 | -63.5 | 108.5 | -63.5 | 0.82 | 2.03 | 0.40 | 0.96 |
| 63.5 | -108.5 | 86 | -86 | 1.40 | 1.30 | 1.08 | 0.92 |
| 63.5 | -108.5 | 86 | -108.5 | 1.60 | 1.60 | 1.00 | 0.91 |
| 63.5 | -63.5 | 108.5 | -86 | 0.91 | 1.90 | 0.48 | 0.93 |
| 63.5 | -86 | 108.5 | -63.5 | 0.98 | 2.02 | 0.48 | 0.95 |
| 86 | -108.5 | 108.5 | -86 | 1.90 | 2.34 | 0.81 | 0.94 |
| 86 | -63.5 | 108.5 | -86 | 1.19 | 2.01 | 0.59 | 0.92 |
| 63.5 | -108.5 | 63.5 | -86 | 1.30 | 0.96 | 1.36 | 0.90 |
| 63.5 | -108.5 | 108.5 | -108.5 | 1.86 | 2.43 | 0.77 | 0.91 |
| 86 | -108.5 | 86 | -63.5 | 1.61 | 1.41 | 1.14 | 0.95 |
| 86 | -108.5 | 63.5 | -108.5 | 1.80 | 1.48 | 1.22 | 0.91 |
| 108.5 | -63.5 | 108.5 | -63.5 | 1.73 | 2.11 | 0.82 | 0.84 |
| 86 | -108.5 | 86 | -86 | 1.78 | 1.55 | 1.15 | 0.96 |
| 63.5 | -108.5 | 108.5 | -86 | 1.50 | 2.10 | 0.71 | 0.90 |
| 86 | -86 | 108.5 | -86 | 1.41 | 2.15 | 0.66 | 0.94 |
| 63.5 | -63.5 | 63.5 | -108.5 | 0.83 | 1.12 | 0.75 | 0.96 |
| 108.5 | -108.5 | 108.5 | -86 | 2.66 | 2.78 | 0.95 | 0.95 |
| 86 | -108.5 | 108.5 | -108.5 | 2.28 | 2.73 | 0.84 | 0.95 |
| 63.5 | -86 | 86 | -63.5 | 0.90 | 1.15 | 0.78 | 0.97 |
| 63.5 | -63.5 | 108.5 | -108.5 | 1.22 | 2.25 | 0.54 | 0.94 |
| 108.5 | -86 | 108.5 | -86 | 2.07 | 2.40 | 0.86 | 0.88 |
| 63.5 | -108.5 | 86 | -63.5 | 1.26 | 1.22 | 1.03 | 0.91 |
| 86 | -108.5 | 86 | -108.5 | 2.01 | 1.88 | 1.07 | 0.94 |
| 86 | -86 | 63.5 | -63.5 | 1.14 | 0.89 | 1.28 | 0.96 |
| 108.5 | -86 | 108.5 | -108.5 | 2.42 | 2.67 | 0.91 | 0.91 |
| 86 | -86 | 63.5 | -86 | 1.23 | 1.05 | 1.18 | 0.96 |
| 86 | -63.5 | 86 | -63.5 | 0.97 | 1.13 | 0.86 | 0.91 |
| 108.5 | -63.5 | 108.5 | -108.5 | 2.13 | 2.35 | 0.91 | 0.88 |
| 108.5 | -86 | 63.5 | -86 | 1.80 | 1.16 | 1.55 | 0.91 |
| 63.5 | -86 | 86 | -86 | 1.03 | 1.27 | 0.82 | 0.97 |
| 108.5 | -63.5 | 108.5 | -86 | 1.79 | 2.07 | 0.87 | 0.83 |
| 63.5 | -86 | 63.5 | -108.5 | 1.04 | 1.21 | 0.86 | 0.91 |
| 63.5 | -63.5 | 86 | -63.5 | 0.69 | 1.04 | 0.66 | 0.96 |
| 86 | -86 | 86 | -63.5 | 1.17 | 1.23 | 0.95 | 0.93 |
| 86 | -108.5 | 108.5 | -63.5 | 1.70 | 2.39 | 0.71 | 0.96 |
| 108.5 | -108.5 | 108.5 | -63.5 | 2.41 | 2.63 | 0.92 | 0.93 |
| 86 | -63.5 | 108.5 | -63.5 | 1.07 | 2.07 | 0.52 | 0.93 |
| 63.5 | -63.5 | 63.5 | -86 | 0.74 | 0.85 | 0.87 | 0.95 |
| 63.5 | -86 | 108.5 | -86 | 1.10 | 2.05 | 0.54 | 0.94 |
| 63.5 | -63.5 | 63.5 | -63.5 | 0.65 | 0.70 | 0.94 | 0.95 |
| 86 | -108.5 | 63.5 | -86 | 1.66 | 1.17 | 1.42 | 0.93 |
| 63.5 | -108.5 | 108.5 | -63.5 | 1.33 | 2.09 | 0.64 | 0.91 |
| 86 | -86 | 86 | -108.5 | 1.52 | 1.73 | 0.88 | 0.96 |
| 108.5 | -108.5 | 63.5 | -63.5 | 2.22 | 1.28 | 1.73 | 0.94 |
| 108.5 | -86 | 86 | -86 | 1.95 | 1.51 | 1.29 | 0.90 |
| 63.5 | -86 | 108.5 | -108.5 | 1.41 | 2.35 | 0.60 | 0.94 |
| 86 | -86 | 108.5 | -63.5 | 1.27 | 2.15 | 0.59 | 0.95 |
| 63.5 | -86 | 86 | -108.5 | 1.22 | 1.61 | 0.75 | 0.95 |
| 86 | -63.5 | 86 | -86 | 1.12 | 1.24 | 0.90 | 0.92 |
| 63.5 | -86 | 63.5 | -63.5 | 0.85 | 0.79 | 1.08 | 0.96 |
| 86 | -86 | 108.5 | -108.5 | 1.75 | 2.49 | 0.70 | 0.93 |
| 108.5 | -63.5 | 86 | -86 | 1.68 | 1.27 | 1.32 | 0.87 |
| 108.5 | -86 | 86 | -108.5 | 2.16 | 1.83 | 1.18 | 0.93 |
| 108.5 | -108.5 | 108.5 | -108.5 | 3.00 | 3.06 | 0.98 | 0.94 |
| 108.5 | -108.5 | 86 | -86 | 2.49 | 1.85 | 1.34 | 0.93 |
| 108.5 | -86 | 63.5 | -63.5 | 1.66 | 1.00 | 1.66 | 0.90 |
| 108.5 | -108.5 | 63.5 | -108.5 | 2.53 | 1.76 | 1.44 | 0.94 |
| 108.5 | -108.5 | 63.5 | -86 | 2.32 | 1.45 | 1.60 | 0.94 |
| 108.5 | -86 | 108.5 | -63.5 | 1.85 | 2.27 | 0.82 | 0.86 |
| 108.5 | -63.5 | 86 | -63.5 | 1.51 | 1.13 | 1.33 | 0.83 |
| 108.5 | -108.5 | 86 | -63.5 | 2.33 | 1.66 | 1.40 | 0.90 |
| 63.5 | -86 | 63.5 | -86 | 0.93 | 0.92 | 1.01 | 0.92 |
| 63.5 | -63.5 | 86 | -86 | 0.82 | 1.17 | 0.70 | 0.93 |
| 108.5 | -63.5 | 63.5 | -63.5 | 1.49 | 0.80 | 1.86 | 0.89 |
| 108.5 | -86 | 86 | -63.5 | 1.75 | 1.37 | 1.28 | 0.87 |
| 86 | -86 | 63.5 | -108.5 | 1.34 | 1.34 | 1.00 | 0.93 |
| 86 | -63.5 | 63.5 | -86 | 1.01 | 0.91 | 1.11 | 0.94 |
| 108.5 | -63.5 | 86 | -108.5 | 1.85 | 1.65 | 1.12 | 0.91 |
| 86 | -63.5 | 63.5 | -63.5 | 0.87 | 0.77 | 1.13 | 0.85 |
| 86 | -63.5 | 63.5 | -108.5 | 1.10 | 1.19 | 0.92 | 0.95 |
| 108.5 | -63.5 | 63.5 | -86 | 1.49 | 0.92 | 1.61 | 0.89 |
| 63.5 | -108.5 | 63.5 | -108.5 | 1.43 | 1.23 | 1.16 | 0.83 |
| 86 | -86 | 86 | -86 | 1.34 | 1.22 | 1.10 | 0.92 |
| 108.5 | -86 | 63.5 | -108.5 | 1.98 | 1.43 | 1.38 | 0.86 |
| 86 | -63.5 | 86 | -108.5 | 1.29 | 1.62 | 0.80 | 0.95 |
| 86 | -108.5 | 63.5 | -63.5 | 1.56 | 1.04 | 1.51 | 0.93 |
| 86 | -63.5 | 108.5 | -108.5 | 1.48 | 2.31 | 0.64 | 0.91 |

**Alternating magnetic field uniformity:**

Figure S11: AMF uniformity map measured within the solenoid coil. Spatial variation remains low over the central region, confirming consistent AMF exposure during heating experiments.

**Schematic of the additive manufactured phantom for verification and validation**

Figure S12: Schematic of the additively manufactured phantom used for the verification and validation experiments. (a) Verification configuration: Eppendorf tubes containing magnetic nanoparticles (MNPs) were placed at vial locations V1-V3. Surface temperatures were measured noninvasively using an infrared camera. (b) Validation configuration: MNPs were placed at vial locations V1-V4, with temperature probes P1-P4 positioned in the corresponding vials. The principal phantom dimensions and spatial arrangement of the vials are indicated. A detailed engineering drawing is provided as a separate supplementary file.

Verification experiments (additional runs):

1. *Run 2:*

Figure S13: Verification of spatially selective heating with a steerable FFR (Run 2). a. Baseline IR image at t = 0 min shows no temperature rise. b. With the AMF applied without FFR selection, all three vials heat. c–e. Steering the FFR to the top (c), right (d), and left (e) vial localizes heating to the targeted vial with only minimal off-target heating in the others. f. Temperature versus time for regions of interest centered on each vial, demonstrates pulsed heating and dynamic FFR steering. The temperature of the targeted vial rises during pulsed heating, while the non-targeted vials remain near baseline. This verifies that the device delivers controllable, spatially confined heating.

2. *Run 3:*

Figure S14: Verification of spatially selective heating with a steerable FFR (Run 3). a. Baseline IR image at t = 0 min shows no temperature rise. b. With the AMF applied without FFR selection, all three vials heat. c–e. Steering the FFR to the top (c), right (d), and left (e) vial localizes heating to the targeted vial with only minimal off-target heating in the others. f. Temperature versus time for regions of interest centered on each vial, demonstrates pulsed heating and dynamic FFR steering. The temperature of the targeted vial rises during pulsed heating, while the non-targeted vials remain near baseline. This verifies that the device delivers controllable, spatially confined heating.

**Pulsed Box-Lucas analysis (additional results):**

1. *Run 1:*

Figure S15: PBL model fits for pulsed heating at 33% duty cycle and 13.04 [kA/m] (Run 1). Panels (a–d) correspond to four independent pulses.

Table S2: SLP at different FFR positions for Run 1 at points P1, P2 and P3 for each pulse.

|  | **Pulse** | **SLP @ P1 [W/g_Fe_]** | **SLP @ P2 [W/g_Fe_]** | **SLP @ P3 [W/g_Fe_]** |
| --- | --- | --- | --- | --- |
| **All heating** | 1 | 38.92 | 60.28 | 60.77 |
|  | 2 | 36.06 | 58.29 | 57.16 |
|  | 3 | 39.03 | 60.84 | 59.25 |
|  | 4 | 39.51 | 59.49 | 58.35 |
| **FFR at P1** | 1 | 62.30 | 22.313 | 21.19 |
|  | 2 | 67.38 | 23.70 | 23.94 |
|  | 3 | 65.31 | 23.55 | 24.00 |
| **FFR at P2** | 1 | 14.48 | 126.17 | 11.86 |
|  | 2 | 15.68 | 146.45 | 13.69 |
|  | 3 | 15.64 | 141.45 | 13.99 |
|  | 4 | 14.15 | 143.13 | 11.03 |
| **FFR at P3** | 1 | 15.09 | 15.51 | 141.03 |
|  | 2 | 21.01 | 21.90 | 115.87 |
|  | 3 | 14.92 | 14.30 | 106.59 |

Table S 3: Mean and standard deviation of SLP at different FFR positions for Run 1 at points P1, P2 and P3.

|  | **SLP @ P1 [W/g_Fe_]** | **SLP @ P2 [W/g_Fe_]** | **SLP @ P3 [W/g_Fe_]** |
| --- | --- | --- | --- |
| **All heating** | 38.38±1.57 | 59.72±1.11 | 58.88±1.52 |
| **FFR at P1** | 65.00±2.56 | 23.19±0.76 | 23.05±1.61 |
| **FFR at P2** | 14.99±0.79 | 139.30±9.00 | 12.65±1.43 |
| **FFR at P3** | 17.02±3.48 | 17.24±4.09 | 121.16±17.82 |

Figure S16: Heating intensity calculated for each pulse using parameters extracted from the PBL fits (Run 1). Panels (a–d) correspond to four independent pulsed-heating datasets.

2. *Run 2:*

Figure S17: PBL model fits for pulsed heating at 33% duty cycle and 13.04 [kA/m] (Run 2). Panels (a–d) correspond to four independent pulses.

Table S4: SLP at different FFR positions for Run 2 at points P1, P2 and P3 for each pulse.

|  | **Pulse** | **SLP @ P1 [W/g_Fe_]** | **SLP @ P2 [W/g_Fe_]** | **SLP @ P3 [W/g_Fe_]** |
| --- | --- | --- | --- | --- |
| **All heating** | 1 | 36.87 | 54.26 | 56.22 |
|  | 2 | 39.08 | 57.76 | 57.75 |
|  | 3 | 41.08 | 60.51 | 59.87 |
|  | 4 | 42.31 | 60.64 | 59.26 |
| **FFR at P1** | 1 | 65.39 | 22.72 | 21.66 |
|  | 2 | 66.94 | 24.08 | 23.59 |
|  | 3 | 67.12 | 24.43 | 23.41 |
| **FFR at P2** | 1 | 12.98 | 133.60 | 10.54 |
|  | 2 | 15.42 | 150.64 | 13.42 |
|  | 3 | 16.37 | 144.23 | 13.29 |
|  | 4 | 16.30 | 146.53 | 13.46 |
| **FFR at P3** | 1 | 14.67 | 14.60 | 128.01 |
|  | 2 | 13.54 | 13.93 | 115.42 |
|  | 3 | 12.05 | 11.14 | 110.77 |

Table S 5: Mean and standard deviation of SLP at different FFR positions for Run 2 at points P1, P2 and P3.

|  | **SLP @ P1 [W/g_Fe_]** | **SLP @ P2 [W/g_Fe_]** | **SLP @ P3 [W/g_Fe_]** |
| --- | --- | --- | --- |
| **All heating** | 39.83±2.38 | 58.29±3.00 | 58.27±1.63 |
| **FFR at P1** | 66.48±0.96 | 23.74±0.90 | 22.88±1.07 |
| **FFR at P2** | 15.26±1.59 | 143.74±7.27 | 12.68±1.43 |
| **FFR at P3** | 13.42±1.32 | 13.22±1.83 | 118.06±8.92 |

Figure S18: Heating intensity calculated for each pulse using parameters extracted from the PBL fits (Run 2). Panels (a–d) correspond to four independent pulsed-heating datasets.

3. *Run 3:*

Figure S19: PBL model fits for pulsed heating at 33% duty cycle and 13.04 [kA/m] (Run 3). Panels (a–d) correspond to four independent pulses.

Table S6: SLP at different FFR positions for Run 3 at points P1, P2 and P3 for each pulse.

|  | **Pulse** | **SLP @ P1 [W/g_Fe_]** | **SLP @ P2 [W/g_Fe_]** | **SLP @ P3 [W/g_Fe_]** |
| --- | --- | --- | --- | --- |
| **All heating** | 1 | 38.90 | 55.75 | 57.77 |
|  | 2 | 39.88 | 57.52 | 57.71 |
|  | 3 | 44.34 | 63.07 | 60.36 |
|  | 4 | 44.20 | 63.07 | 60.84 |
| **FFR at P1** | 1 | 68.08 | 23.26 | 21.13 |
|  | 2 | 76.74 | 26.79 | 24.83 |
|  | 3 | 81.35 | 26.90 | 25.67 |
| **FFR at P2** | 1 | 12.83 | 133.63 | 10.57 |
|  | 2 | 18.31 | 156.67 | 15.03 |
|  | 3 | 14.45 | 146.39 | 12.79 |
|  | 4 | 14.67 | 144.80 | 11.79 |
| **FFR at P3** | 1 | 14.64 | 14.61 | 119.60 |
|  | 2 | 13.65 | 13.93 | 112.50 |
|  | 3 | 12.75 | 13.46 | 113.68 |

Table S 7: Mean and standard deviation of SLP at different FFR positions for Run 3 at points P1, P2 and P3.

|  | **SLP @ P1 [W/g_Fe_]** | **SLP @ P2 [W/g_Fe_]** | **SLP @ P3 [W/g_Fe_]** |
| --- | --- | --- | --- |
| **All heating** | 41.83±2.85 | 59.85±3.78 | 59.17±1.66 |
| **FFR at P1** | 75.39±6.74 | 25.65±2.07 | 23.88±2.41 |
| **FFR at P2** | 15.06±2.31 | 145.37±9.43 | 12.54±1.89 |
| **FFR at P3** | 13.68±0.95 | 14.00±0.58 | 115.26±3.80 |

Figure S20: Heating intensity calculated for each pulse using parameters extracted from the PBL fits (Run 3). Panels (a–d) correspond to four independent pulsed-heating datasets.

**Validation experiment results (pulsed heating at 33% duty cycle):**

1. *Run 1:*

Figure S21: Pulsed heating results in a liver phantom (Run 1). a. Coil-bore view with probe locations (P1–P4). b. Applied AMF amplitude showing 45 [s] ON and 90 [s] OFF cycles. Heating is paused during FFR switching. c. Temperature rise at each probe: localized heating at the targeted tumor-mimicking sites in sequence with minimal rise at P4 (healthy tissue).

2. *Run 2:*

Figure S22: Pulsed heating results in a liver phantom (Run 2). a. Coil-bore view with probe locations (P1–P4). b. Applied AMF amplitude showing 45 [s] ON and 90 [s] OFF cycles. Heating is paused during FFR switching. c. Temperature rise at each probe: localized heating at the targeted tumor-mimicking sites in sequence with minimal rise at P4 (healthy tissue).

3. *Run 3:*

Figure S23: Pulsed heating results in a liver phantom (Run 3). a. Coil-bore view with probe locations (P1–P4). b. Applied AMF amplitude showing 45 [s] ON and 90 [s] OFF cycles. Heating is paused during FFR switching. c. Temperature rise at each probe: localized heating at the targeted tumor-mimicking sites in sequence with minimal rise at P4 (healthy tissue).

**Validation experiment additional results (autotuning PID heating):**

1. *Run 2:*

Figure S24: Autotuning PID validation in the liver phantom (scenario 1, Run 2: P1–P3 tumor, P4 healthy tissue). a. Coil-bore view of the liver phantom showing fiber-optic probe locations P1–P4. b. Applied AMF amplitude versus time, with the initial autotuning phase followed by the tuned phase; the dashed line indicates the maximum field amplitude of 13.04 [kA/m]. c. Temperature trajectories at each probe: as the FFR is steered between targets, P1–P3 track plateaus near the 43.5 [°C] setpoint, whereas P4 remains several degrees cooler. d. Corresponding CEM43 dose accumulation, demonstrating therapeutic dose (>8 [min]) at P1–P3 and negligible dose at the healthy-tissue probe P4.

2. *Run 3:*

Figure S25: Autotuning PID validation in the liver phantom (scenario 1, Run 3: P1–P3 tumor, P4 healthy tissue). a. Coil-bore view of the liver phantom showing fiber-optic probe locations P1–P4. b. Applied AMF amplitude versus time, with the initial autotuning phase followed by the tuned phase; the dashed line indicates the maximum field amplitude of 13.04 [kA/m]. c. Temperature trajectories at each probe: as the FFR is steered between targets, P1–P3 track plateaus near the 43.5 [°C] setpoint, whereas P4 remains several degrees cooler. d. Corresponding CEM43 dose accumulation, demonstrating therapeutic dose (>8 [min]) at P1–P3 and negligible dose at the healthy-tissue probe P4.

**References:**

S1. Carlton, H., & Ivkov, R. (2023). A new method to measure magnetic nanoparticle heating efficiency in non-adiabatic systems using transient pulse analysis. Journal of Applied Physics, 133(4).
